# 3D bioprinting of engineered exosomes secreted from M2-polarized macrophages through immunomodulatory biomaterial promotes *in vivo* wound healing and angiogenesis

**DOI:** 10.1016/j.bioactmat.2024.11.026

**Published:** 2024-11-27

**Authors:** Sayan Deb Dutta, Jeong Man An, Jin Hexiu, Aayushi Randhawa, Keya Ganguly, Tejal V. Patil, Thavasyappan Thambi, Jangho Kim, Yong-kyu Lee, Ki-Taek Lim

**Affiliations:** a Department of Biosystems Engineering, Kangwon National University, 24341, Chuncheon, Republic of Korea; b Institute of Forest Science, Kangwon National University, 24341, Chuncheon, Republic of Korea; c School of Medicine, University of California Davis, 95817, Sacramento, United States; d Department of Bioengineering, College of Engineering, Hanyang University, 04763, Seoul, Republic of Korea; e Department of Plastic and Traumatic Surgery, Capital Medical University, 100069, Beijing, China; f Interdisciplinary Program in Smart Agriculture, Kangwon National University, 24341, Chuncheon, Republic of Korea; g Graduate School of Biotechnology, College of Life Sciences, Kyung Hee University, 17104, Yongin, Republic of Korea; h Department of Convergence Biosystems Engineering, Chonnam National University, 61186, Gwangju, Republic of Korea; i Department of Chemical and Biological Engineering, Korea National University of Transportation, 27470, Chungju, Republic of Korea

**Keywords:** Exosome, Immunomodulation, Decellularized extracellular matrix, Skin bioprinting, Angiogenesis, Wound healing

## Abstract

Biomaterial composition and surface charge play a critical role in macrophage polarization, providing a molecular cue for immunomodulation and tissue regeneration. In this study, we developed bifunctional hydrogel inks for accelerating M2 macrophage polarization and exosome (Exo) cultivation for wound healing applications. For this, we first fabricated polyamine-modified three-dimensional (3D) printable hydrogels consisting of alginate/gelatin/polydopamine nanospheres (AG/NSPs) to boost M2-exosome (M2-Exo) secretion. The cultivated M2-Exo were finally encapsulated into a biocompatible collagen/decellularized extracellular matrix (COL@d-ECM) bioink for studying angiogenesis and *in vivo* wound healing study. Our findings show that 3D-printed AGP hydrogel promoted M2 macrophage polarization by Janus kinase/signal transducer of activation (JAK/STAT), peroxisome proliferator-activated receptor (PPAR) signaling pathways and facilitated the M2-Exo secretion. Moreover, the COL@d-ECM/M2-Exo was found to be biocompatible with skin cells. Transcriptomic (RNA-Seq) and real-time PCR (qRT-PCR) study revealed that co-culture of fibroblast/keratinocyte/stem cells/endothelial cells in a 3D bioprinted COL@d-ECM/M2-Exo hydrogel upregulated the skin-associated signature biomarkers through various regulatory pathways during epidermis remodeling and downregulated the mitogen-activated protein kinase (MAPK) signaling pathway after 7 days. In a subcutaneous wound model, the 3D bioprinted COL@d-ECM/M2-Exo hydrogel displayed robust wound remodeling and hair follicle (HF) induction while reducing canonical pro-inflammatory activation after 14 days, presenting a viable therapeutic strategy for skin-related disorders.

## Highlights

• We propose the development of new hydrogel inks for 3D encapsulation of M2 macrophage-derived exosomes and bioprinting of human skin cells with an aim to accelerate *in vivo* wound healing and angiogenesis.
• The catecholamine-modified alginate/gelatin hydrogels are utilized for the macrophage polarization and cultivation of M2 exosomes from RAW 264.7 cells.
• The d-ECM bioink with skin cells and exosomes displayed vascularized wound healing via mechanical/cytokine-associated signaling pathways.

## Graphical abstract

**Figure:**
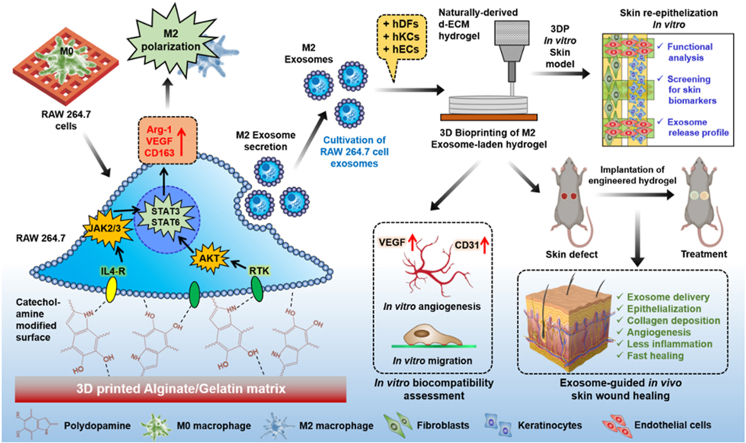

## 1 Introduction

Wound healing is a dynamic process involving a series of signaling pathways connected to pre-inflammation, post-healing, hemostasis, cell proliferation, cell migration, and epidermal remodeling [1]. A pronounced shift in the inflammatory signaling pathway is crucial for determining the fate of wound healing and fibrotic scar formation. Among the various wound healing processes, cutaneous wound healing is a complex biological process that involves the activation of several growth factors, cytokines, and chemokines to repair damaged tissue, thereby conferring protection against external damage [[2], [3], [4]]. Cutaneous wounds are associated with various clinical conditions, such as acute trauma, severe burns, diabetes, accidental injury, or cuts with delayed angiogenesis. This creates a huge socioeconomic burden [5]. A rationally designed strategy could address such clinical wound-related problems. Immune cells, specifically macrophages, play a critical role in wound healing via activation and differentiation of various inflammatory phenotypes, such as pro-inflammatory (M1) or anti-inflammatory (M2) phenotypes at the infected or inflammation sites. Among the various immune cells, macrophages are a major regulatory type involved in the wound healing process [[6], [7], [8]]. In the early stage of wound healing (0–3 days), the macrophages polarized into the M1 phenotype to remove dead cells and combat against pathogens. After 5–7 days of pre-healing, macrophages display the M2 phenotype, which aids in tissue repair and regeneration via secretion of various anti-inflammatory factors, such as interleukin-4 (IL-4), interleukin-10 (IL-10) and transforming growth factor-β (TGF-β), that result in wound healing [7,9]. Therefore, the wound-healing process is largely determined by the balance between the proportion of M1 and M2 macrophages.

It has been shown that biomaterial properties, such as surface charge, functional groups, wettability, embedded nanoparticles (metal or non-metal), and mechanotopography, significantly influence the polarization of macrophages during wound repair and regeneration. For instance, aminated (-NH_2_), carboxylated (-COO^-^), and sulfonate (-SO_3_H) biomaterials promoted early expression of the M1 phenotype while displaying an M2 phenotype in murine monocytes (RAW 264.7) during tissue regeneration [5,10,11]. Several nanocomposite hydrogels functionalized with NH_2_-polystyrene nanoparticles (PNPs), polydopamine-iron oxide (PDA-Fe_3_O_4_) NPs, silver (Ag) NPs, and metal-organic frameworks (MOFs) exhibited net positive charge onto the surface, which helped in macrophage podosome and filopodia adhesion, and subsequent immunopolarization [[12], [13], [14], [15]]. Naturally derived hydrogels are also a good source for attenuating the fibrotic scar and accelerating wound healing. PDA, a catecholamine derived from mussel foot, holds tremendous potential in macrophage polarization and skin regeneration. Owing to its positively charged nature, the PDA is often used as a coating substance for various biomaterials to improve the hemostatic and wound healing properties [16]. PDA encapsulated in alginate (Alg) and gelatin (Gel)-based hydrogels have been shown to promote fast wound healing via inducing angiogenesis and M2 macrophage polarization [[17], [18], [19]]. Similarly, decellularized extracellular matrix (d-ECM)-based hydrogels with tunable mechanical properties (∼100 kPa–300 kPa) have been reported to enhance the M2 macrophage polarization and wound healing by inducing the fibroblast migration and VEGF secretion [20]. Thus, the selection of suitable biomaterials and/or nanocomposites is crucial for regulating macrophage polarization and subsequent wound healing.

Recently, exosomes (Exo) derived from mesenchymal and epithelial cells demonstrated outstanding therapeutic advances in cutaneous wound healing owing to their capacity to deliver various proteins and soluble factors directly into the wound bed. Great efforts have been made to develop Exo-based scaffolds/hydrogels for skin regeneration [21]. 3D bioprinting, an innovative additive manufacturing (AM) technology, holds tremendous potential for skin tissue engineering owing to its ability to print layer-by-layer 3D structures with high accuracy. Most bioprinted *in vitro* or *in vivo* skin models rely on the direct or indirect manipulation of skin cells without the involvement of Exos. Thus far, most reported bioprinted Exo-based platforms have been used for bone or cartilage tissue engineering [22]. For instance, various multimaterial bioinks, such as alginate/gelatin, methacrylated hyaluronic acid (HA-MA)/gelatin methacrylate (GelMA), and methacrylated silk fibroin (SF-MA)/HA-MA/Cu has been shown to exhibit skin cells growth and re-epithelialization via inducing fibroblast migration and angiogenesis [[23], [24], [25], [26]]. Furthermore, dermal and epidermal spheroids printed with endothelial cells in a collagen-based hydrogel showed rapid regeneration of skin wounds with accelerated hair follicle (HF) formation [27]. A comparative study of the available AM technology for skin bioprinting is presented in Table S1. Despite great advancements in skin bioprinting, a few reports in the literature have demonstrated macrophage Exo-laden hydrogels for wound healing applications. Geng et al. reported that the use of hMSC-derived Exo-incorporated carboxymethyl chitosan/dialdehyde carboxymethyl cellulose scaffolds promoted M2 macrophage polarization and accelerated wound healing through rapid proliferation and neo-angiogenesis of endothelial cells [28]. Han et al. recently reported that BSA-conjugated Exo-loaded nanobubble (EBO)/gelatin/polyvinyl alcohol hydrogel promoted traumatic wound healing via inducing rapid hemostasis [29]. Yang and co-workers [30] also demonstrated that hMSC-derived Exo-laden hyaluronic acid hydrogel showed robust wound healing via sustained release of miR-21-5p. Interestingly, using M2-Exo and AuNR-loaded polyacrylamide (PANB/Au/AC/Exo) hydrogel, Li et al. reported a photothermal platform capable of attenuating infected wounds via enhanced antimicrobial properties [31]. These reports are some outstanding examples of Exo-laden hydrogel platforms for skin regeneration. However, the role of macrophage-derived Exo and their 3D bioprinting with skin cells for wound healing is still unknown. Most fabricated hydrogel platforms deliver Exo, where the release behavior of Exo is either slow or restricted due to the hydrogel's tough crosslinking. Thus, a therapeutic bioink consisting of M2-Exo and skin cells would enable better uptake of M2-Exo to the cells, which upon transplantation would promote robust wound healing *in vivo*.

**Scheme 1:**
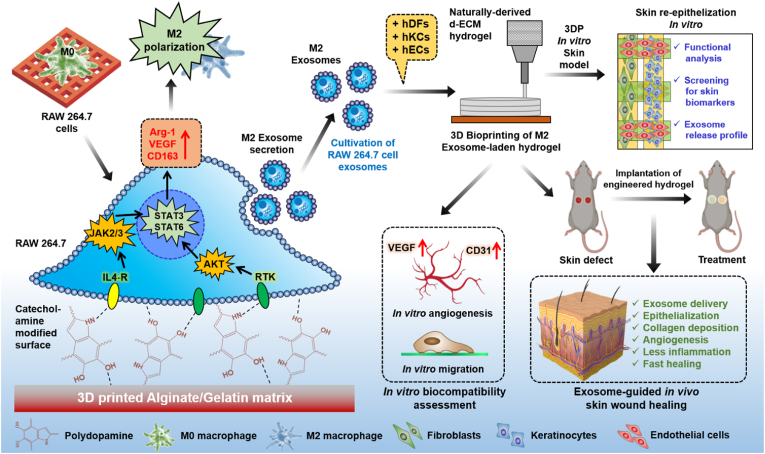
Schematic illustration of the bioink development for anti-inflammatory exosome production and 3D bioprinting towards skin regeneration.

Considering that hydrogel composition greatly influences M2 macrophage polarization and Exo secretion, we developed a pair of bioinks for enhancing Exo secretion from M2 polarized monocyte/macrophages and 3D bioprinting of skin cells (Scheme 1). First, we fabricated a catecholamine-based bioink (bioink-I) composed of alginate/gelatin/polydopamine nanospheres (Alg/Gel/PDA NSPs) to facilitate macrophage adhesion, proliferation, and polarization. Next, we developed a collagen/skin-derived decellularized extracellular matrix (COL@d-ECM-mExo-AGP) bioink (bioink-II) for 3D skin printing using human dermal fibroblasts (hDFs), keratinocytes (hKCs), stem cells (hMSCs), and endothelial cells (hECs). The fabricated skin bioink exhibited typical shear-thinning properties and exhibited remarkable shape fidelity during bioprinting. A Pluronic®-based self-healing supporting bath gel (SBG) was used for skin bioprinting. We demonstrated that M2 polarized macrophage-derived Exos (mExo-AGP) showed higher stability, probe binding affinity, and excellent metabolic activity in cultured cells compared to the control. Furthermore, mExo-AGP exhibited greater angiogenic capacity in the presence of hMSCs and hECs. The bioprinted skin construct with mExo-AGP showed a sustained release of exosomes during cell culture, which could be beneficial for complete skin regeneration. The *in vivo* wound healing study of 3D printed COL@d-ECM + Exo hydrogel further demonstrated excellent biosafety and accelerated wound healing via collagen deposition and reduced inflammation. In summary, we showed that our fabricated skin bioink is non-toxic, highly biocompatible, and displays controllable release behavior of M2-Exos. Thus, we hope that our efforts to improve skin regeneration will provide a path for scientists to fabricate next-generation smart skin grafts for treating skin-related injuries in clinical settings.

## 2 Experimental section

### 2.1 Cell culture

The human dermal fibroblast (hDFs, PCS-201-012, ATCC®) cells were cultured in Dulbecco's Modified Eagle Medium (DMEM)/Hams-F12 (1:1) media (Welgene, Republic of Korea) containing 10 ng mL^−1^ basic fibroblast growth factor (b-FGF) with 10 % fetal bovine serum (FBS) and 1 % penicillin/streptomycin (P/S). The human keratinocyte (HaCaT, PCS-200-011, ATCC®) cells cultured in DMEM/Hams-F12 (3:1) media supplemented with 0.5 mM L^−1^ L-ascorbic acid, 2.5 μg mL epidermal growth factor (EGF), 2 % FBS, and 1 % P/S. The human bone marrow-derived mesenchymal stem cells (hBMSCs, 499Z010, PromoCell) were culture DMEM containing 10 % FBS and 1 % P/S. The endothelial cells (hECs, PCS-100-013, ATCC®) were cultured in endothelial growth media (EGM-2, Promo Cell, Heidelberg, Germany) supplemented with 0.02 mL fetal calf serum (FCS), 5 ng mL^−1^ FGF, 10 ng mL^−1^ b-FGF, 20 ng mL^−1^ insulin growth factor-1 (IGF-1), 0.5 ng mL^−1^ vascular endothelial growth factor-165 (VEGF-165), 1 μg mL^−1^ L-ascorbic acid, 22.5 μg mL^−1^ Heparin, and 0.2 μg mL^−1^ hydrocortisone, respectively. For neurogenic differentiation, the hBMSCs were cultured in DMEM containing neurogenic supplements (StemPro, Thermo-Fischer Scientific, USA). The murine macrophage (RAW 264.7, 40071, KCLB) cells were cultured in DMEM media containing 10 % FBS and 1 % P/S. The growth factors and 1 × PBS were obtained from Sigma-Aldrich, USA.

### 2.2 Bioink fabrication

Two types of bioinks were formulated in this study. An alginate/gelatin/PDA NSP bioink was developed for macrophage phenotyping and exosome extraction. For skin bioprinting, COL@d-ECM/M2-Exo bioink was fabricated. The details of the bioink preparation are as follows:

#### 2.2.1 Bioinks for macrophage polarization (Group-I bioinks)

For macrophage phenotyping and exosome isolation, we first developed a composite bioink consisting of alginate (A), gelatin (G), and polydopamine nanospheres (PDA NSPs). The concentrations of A and G were chosen, as reported in our previous study [32]. Briefly, 3 % (*w/v*) alginate (Sigma-Aldrich, USA) was dissolved in sterile deionized (DI) water at 65 °C with vigorous stirring. Subsequently, the temperature was lowered to 45 °C, and 2 % (*w/v*) gelatin (porcine Type-B, Sigma-Aldrich, USA) was added to the alginate solution and stirred until fully dissolved. Next, PDA NSPs were added to the alginate/gelatin pre-gel solution with respect to the weight of the polymers (for example, 0.025, 0.05, and 0.075 wt %). The resultant nanocomposite solution was stirred overnight at 45 °C to obtain a homogenous solution. The pre-gel solution was poured into the respective printing cartridges (Cellink, Sweden) and stored at 4 °C until use. The bioinks were designated as AG, AGP-1, AGP-2, and AGP-3 (categorized as Group-I bioinks).

#### 2.2.2 Bioinks for skin printing (Group-II bioinks)

Group-II bioinks were composed of collagen type-1 (COL; Sigma-Aldrich, USA) and chicken skin-derived decellularized extracellular matrix (d-ECM). The decellularization process was conducted as previously reported, with some modifications [33,34]. Fresh chicken skin samples were collected from a local market in Chuncheon, Republic of Korea. The fat layer was then carefully trimmed with a sterile surgical knife and washed several times with 1X PBS. Next, the skin was cut into small pieces and incubated with 10 % sodium dodecyl sulfate (SDS; Sigma-Aldrich, USA) and 5 % Triton-X 100 (Sigma-Aldrich, USA) for 24 h under constant magnetic stirring (150 rpm). After 24 h, the treated skin samples were washed with PBS and further stirred (150 rpm) with DI water for 3 days to remove SDS. Next, the samples were incubated with 0.25 % trypsin-EDTA solution (Welgene, Republic of Korea) and 100 U mL^−1^ DNase (Life Technologies, Thermo Scientific, USA) supplemented with 10 mM MgCl_2_ (Sigma-Aldrich, USA) for 24 h (150 rpm, 37 °C) to digest the ECM and remove traces of nucleic acids. After enzymatic digestion, d-ECM was washed with PBS for at least 2–3 days to remove residual trypsin and DNases. The samples were then lyophilized, yielding a white foam-like structure. After the pretreatment process, the material was used for the preparation of a hybrid bioink. For bioink preparation, d-ECM (500 mg) and COL (50 mg) were dissolved in 0.5 M acetic acid (Daejung Chemicals, Republic of Korea) and pepsin-HCl (10 mg/100 mg of d-ECM) solution and gently stirred until fully dissolved. We observed a thick paste-like pre-gel mixture after pepsin-HCl treatment. Once the components were fully dissolved, the pH of the mixture was adjusted to 7.4 using 10 M NaOH (Fischer Chemicals, USA). The pregel mixture was loaded onto a printing cartridge and stored at 4 °C until further use. Bioinks were designated as COL, COL@d-ECM, and COL@d-ECM/M2-Exo (where Exo was loaded), respectively.

### 2.3 3D bioprinting

The 3D printing and bioprinting were conducted using an extrusion-based 3D bioprinter coupled with a pneumatic print head and a temperature-controlled print bed. All the 3D structures were designed using SolidWorks software (Dassault Systems, France). The 3D bioprinting was carried out using BIO-X provided by CELLINK Corporation, Sweden. Detailed information on printing parameters is listed in Fig. S3, respectively.

### 2.4 Characterizations

The morphology of the PDA NSPs was investigated using FE-SEM and HR-TEM with an acceleration voltage of 15 kV cm^−1^. The fabricated hydrogel scaffold's chemical interaction and structural features were evaluated using FT-IR (400–4000 cm^−1^, 10 cm^−1^ scan rate) and XRD (diffraction range 2θ = 10–40°) spectroscopy. The scaffold morphology was investigated using an FE-SEM (Jeol, Japan) instrument with an operating voltage of 15 kV cm^−1^ in LED mode. A rotational rheometer (Anton Parr, Austria) with an 8 mm parallel plate was used to study the viscoelastic properties of the developed hydrogel inks. The hydrogels were measured through flow and temperature sweeps with an angular frequency of 0.1–100 Rad s^−1^ and a shear rate of 0.1–100 s^−1^, respectively. The swelling efficiency of the developed hydrogel scaffolds was assessed, as reported in our previous study [32]. The exosome release kinetics and time-resolved fluorescence decay were studied using a fluorescence spectrophotometer (Spectramax M, Molecular Devices, USA).

### 2.5 Macrophage polarization study

The macrophage polarization potential was investigated using Group-I bioinks by Quanti-Seq 3′ RNA sequencing (RNA-Seq), fluorescent activated cell sorting (FACS), immunocytochemistry (ICC), and quantitative real-time polymerase chain reaction (qRT-PCR) analysis, respectively. The RAW 264.7 cell (4 × 10^4^ cells/24-well/mL) culture was performed using a 10 × 5 mm^2^ (infill density: 25 %) 3D printed hydrogel for all experiments. For RNA-Seq, the 3D printed hydrogel scaffolds were crosslinked with 100 mM CaCl_2_ (Sigma-Aldrich, USA) solution post-printing and incubated with DMEM medium for 24 h. After 24 h, the hydrogels were washed with 1 × PBS and incubated with RAW 264.7 cells (4 × 10^4^/1 mL of media) for 24 h in a humidified atmosphere containing 5 % CO_2_. After 24 h, the total RNA was harvested using RNAzol RT reagent (Sigma-Aldrich, USA), and the RNA purity was quantified using a Nanodrop spectrophotometer (TECAN Pro, Switzerland). Next, the Quanti-Seq 3′ mRNA-Seq was conducted using a next-generation sequencer (NGS, Nova-Seq 6000, PE100 bp, California, USA) using the reference genome mm10 and genome database UCSC for the mouse. The raw data were screened using an Excel-based differentially expressed gene analysis (ExDEGA) graphic software (ebiogen, Republic of Korea), and the data were normalized to Log2 fold. The student's *t*-test was conducted to study the significant changes in the mRNA expression. Additionally, the iDEP.96 software (South Dakota State University, USA) and Gene Set Enrichment Analysis (GSEA) were performed to visualize the enrichment in various groups. The differentially expressed genes (DEGs) with a fold change of ≥2.0 and a *P* value < 0.05 were considered statistically significant.

Additionally, the macrophage polarization potential was also verified using flow cytometry against NOS2 (M1 phenotype) and CD163 (M2 phenotype) markers after 24 h of incubation. For ICC analysis, the RAW 264.7 cells (2.5 × 10^4^ growing onto the 3D printed hydrogels) were fixed with 3.7 % PFA (Sigma-Aldrich, USA) for 15 min, followed by permeabilized with ice-cold methanol (100 %) for 5 min. After that, the cells were blocked with 1 % BSA (Sigma-Aldrich, USA) solution for 1 h. After blocking, the cells were incubated with Fc receptor blocker for 30 min, followed by incubation with primary antibodies against NOS2 (M1 marker), CD163 (M2 marker), and VEGFA (M2 marker) for 1 h at room temperature. Next, the cells were washed with 1 × PBS and incubated with fluorescent secondary antibody tagged with FITC (ex: 488/519 nm) and rhodamine dyes. The nucleus was counterstained with DAPI (Sigma-Aldrich, USA). After desirable staining, the cells were mounted with suitable mounting media and visualized using an inverted fluorescence microscope (DMi8, Leica, Germany) with appropriate filters. All the antibodies for flow cytometry and ICC were purchased from Santa Cruz Biotechnology, USA, and the dilution factor is given in Table S2.

For qRT-PCR analysis, 24 h grown RAW 264.7 cells (4 × 10^4^ growing onto the 3D printed hydrogels) were treated with 1 × PBS, and the total RNA was harvested and converted into cDNA using a cDNA synthesis kit (Thermo-Fischer Scientific, USA) according to the manufacturer's guidelines. After that, the mRNA expression was quantified using a CFX Maestro Real-time system (Bio-Rad, USA). The relative expression of the pro- and anti-inflammatory genes was quantified using the delta-CT method. The gene primers were purchased from Bioneer Corp., Daejeon, Republic of Korea. The list of gene primers used for qRT-PCR analysis in RAW 264.7 cells is given in Table S3.

### 2.6 M2-exosome (M2-Exo) isolation and characterization

Based on the RNA sequencing, ICC, FACS, and qRT-PCR data, we have selected the AG and AGP-3 hydrogel scaffolds for Exo cultivation from RAW 264.7 cells. We cultivated the M2-Exo using the precipitation method. The details of the exosome isolation, characterization, and *in vitro* bioactivity evaluation are given in the Supporting **Methods** section.

### 2.7 Supporting bath-assisted 3D bioprinting of multi-layered skin construct

#### 2.7.1 Formulation of supporting bath hydrogel (SBG)

We used an SBG for 3D bioprinting of the multilayered skin. The SBG was fabricated using a thermoresponsive Pluronic® (poloxamer 407 or P407, Sigma-Aldrich, USA) gel. We chose 15 % (*w/v*) Pluronic® owing to its superior gelation and self-healing properties during 3D bioprinting. Briefly, 15 % P407 was dissolved in either PBS or MEM in a low-temperature environment. After complete dissolution, the P407 pre-gel solution was poured into plastic petri plates and incubated at 37 °C.

#### 2.7.2 SBG-assisted 3D bioprinting

After gelation, P407 was used as the supporting bath medium for bioprinting the COL@d-ECM gel. Prior to bioprinting, the hMSCs, hDFs, and hKCs were pre-stained with cell-tracking dyes, such as 8-chloromethyl-4,4-difluro-1,3,5,7-tetramethyl-4-bora-3,4-diaza-S-indacene (BODIPY, Thermo-Fisher Scientific, USA), CellTracker Deep Red (Thermo Scientific, USA), and 2,3,6,7-tetrahydro-9-bromomethyl-1H, 5H-quinolizino (9,1-gh) coumarin (Thermo Scientific, USA). After suitable labeling, the cells were mixed with the COL@d-ECM pre-gel solution to achieve a final cell density of 5 × 10^4^. Next, the cell-laden bioinks were loaded onto printing cartridges and incubated at 37 °C for 15 min to ensure gelation. Subsequently, the cell-laden bioinks were installed in the bioprinter (Cellink Bio-X, Sweden), and 3D bioprinting was conducted at varying printing speeds to test filament formation. A 10 × 10 × 5 mm^3^ construct with an infill density of 20 % was used for skin graft bioprinting. A 25G metal-head nozzle (inner diameter: 250 μm; height: 0.5′) was used for filament extrusion testing. The print-bed temperature was set to 37 ± 2 °C to maintain the SBG in gelled form, and the printing speed was set to 5.5 mm s^−1^. After 3D bioprinting, the printed construct was removed from the SBG and rinsed with sterile PBS to remove any residual traces of the SBG. For exosome-laden printing, the same process was repeated with the addition of M2-Exo, and the bioink was named COL@d-ECM/M2-Exo. For cell culture experiments, the bioprinted construct was maintained in various growth and differentiation media for up to 7 days. The detailed composition and list of culture media are shown in Fig. 3(j). The cytotoxicity of the bioprinted construct was evaluated using the WST-8 assay (Cellrix, Republic of Korea) after 7 days of culture. A blood biocompatibility assay (hemolysis test) was conducted to evaluate the *ex vivo* biosafety of the formulated bioinks.

#### 2.7.3 Functional analysis of the bioprinted skin construct

Functional analysis and skin re-epithelialization w/or w/o Exo were evaluated through bulk RNA sequencing. To verify the expression of various dermal, epidermal, and angiogenic gene markers, qRT-PCR was conducted after 7 days of bioprinting and *in vitro* cell culture. Detailed information on gene markers for hDFs, hKCs, and hMSCs is given in Table S4.

#### 2.7.4 Histological evaluation of the bioprinted skin model

To investigate the skin regeneration potential of the developed bioink, we further analyzed the tissue formation ability by hematoxylin and eosin (H&E) and immunohistochemical (IHC) staining, respectively. For H&E staining, the bioprinted skin constructs were fixed with 3.7 % paraformaldehyde and frozen in liquid nitrogen (−196 °C). After that, the frozen tissue was sectioned using a microtome machine with a thickness of 50 μm. Next, the sections were mounted into glass slides and dried in a vacuum oven at 65 °C. Afterward, the slides were rinsed with Hematoxylin (Sigma-Aldrich, USA) solution for 3–5 min, followed by washing with tap water for 2 min. The washed slides were dipped in acid-alcohol (1 %) solution for 2 min and immersed in Eosin-Y (Sigma-Aldrich, USA) solution for 1 min. Next, the slides were washed with tap water and dehydrated in ethanol series (60 %, 70 %, 90 %, and 100 %) each 2 min, followed by three changes of xylene. The stained and dehydrated slides were finally mounted with standard mounting media with coverslips and visualized using an upright optical microscope. For IHC staining, the fixed slides were incubated with primary antibody against CD31 (Santa Cruz Biotechnology, USA), followed by HRP-conjugated secondary antibodies, and stained with a DUB substrate kit (Sigma-Aldrich, USA). The expression of CD31 was photographed and semi-quantified using NIH ImageJ software.

### 2.8 *In vivo* studies

Based on the outstanding *in vitro* results, we further conducted the *in vivo* wound healing assay to study the regenerative ability of the COL@d-ECM and COL@d-ECM + Exo hydrogels. The *in vivo* wound healing was performed using 3–4 weeks old ICR male rates. All animal procedures were conducted and approved by the Institutional Animal Care and Use Committee (IACUC), Capital Medical University, Beijing, China. Details of the *in vivo* surgery, scaffold implantation, staining, and macrophage-mediated wound healing study are provided in the Supporting Methods section.

### 2.9 Image analysis

The bright field and fluorescence images were analyzed using ImageJ software (v1.8, NIH, Bethesda, USA). The cell migration study was quantified using the Cell Counter tool of ImageJ using proper macrocodes. The cell aspect ratio was calculated using ImageJ in terms of length and width. The *in vitro* and *in vivo* histological and immunocytochemical images were processed with ImageJ software, and the total staining area was calculated using the Area Distribution tool. All the image-based experiments were conducted in three biological replicates (*n* = 3) unless stated elsewhere.

### 2.10 Statistical analysis

The statistical analysis was carried out using Origin Pro software (v9.0, Origin Labs, USA). One-way analysis of variance (ANOVA) was carried out to verify the significant difference between the control and treatment groups. Statistical significance was set at ∗*p* < 0.05, ∗∗*p* < 0.01, and ∗∗∗*p* < 0.001. Data are reported as mean ± s.d. of triplicate (*n* = 3) experiments. The three dots in each bar diagram represent the median and interquartile range of each data.

## 3 Results and discussion

### 3.1 3D printed AGP hydrogel promoted M2 macrophage polarization

**Fig. 1:**
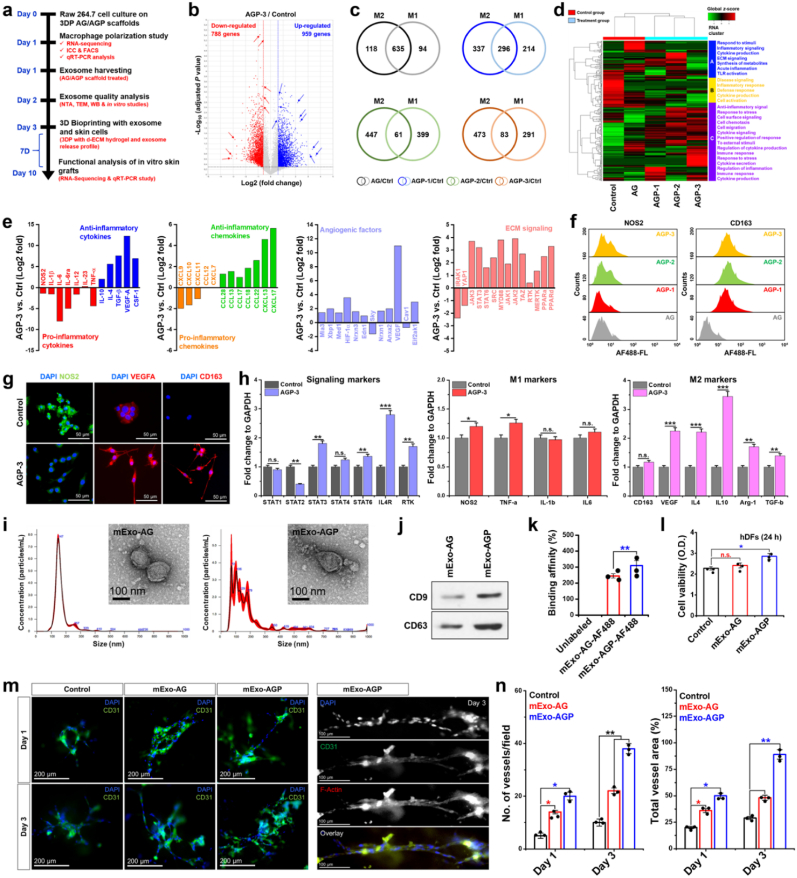
Analysis of macrophage polarization onto the 3D printed polydopamine-modified alginate/gelatin (AGP) scaffolds and exosome cultivation for therapeutic applications. **(a)** Schematic illustration of the immunopolarized exosome cultivation, encapsulation, and 3D bioprinting of functional skin constructs. **(b)** Volcano plot showing the major down or up-regulated genes (Log2 fold, ∗*p* < 0.05) in AGP-3 *vs.* control samples after 24 h of incubation. **(c)** Representative Venn diagrams of the various treatment groups showing the significant M1 and M2 genes as evaluated by RNA-seq. **(d)** Two-way hierarchical clustering of the differentially expressed genes (DEGs) associated with the immune response of macrophages. **(e)** Representative DEGs associated with inflammatory cytokines and chemokines production, angiogenesis, and ECM signaling (Log2 fold, *p* < 0.05) in the presence of AGP-3 scaffold. **(f)** Flow cytometry analysis showing the NOS2 (M1) and CD163 (M2) markers after 24 h of incubation with scaffolds. **(g)** FL images showing the expression of NOS2, VEGFA, and CD163 markers after 24 h of culture with AG (control) and AGP-3 scaffolds. Scale bar: 50 μm. **(h)** qRT-PCR analysis of the major signaling (*STAT1*, *STAT2*, *STAT3*, *STAT4*, *STAT6*, *IL-4R*, and *RTK*), M1 (*NOS2*, *TNF-α*, *IL-1β*, and *IL-6*), and M2 (*CD163*, *VEGF*, *IL-4*, *IL-10*, *Arg-1*, and *TGF-β*) gene markers under scaffold treatment (24 h), Statistical significance at ∗*p* < 0.05, ∗∗*p* < 0.01, and ∗∗∗*p* < 0.001 (One-way ANOVA test). **(i)** NTA analysis with corresponding HR-TEM morphology of the mExo-Ctrl (AG) and mExo-AGP (AGP). **(j)** Representative western blotting of exosomes showing the presence of CD9 and CD63. **(k)** The FITC binding affinity of the exosomes. **(l)** WST-8 assay showing the *in vitro* cytotoxicity profile of the exosomes in hDFs. **(m)***In vitro* angiogenic potential of the hECs in the presence of exosomes at indicated time points. **(n)** The relative quantification data of the angiogenesis assay. Data reported are mean ± s.d. of triplicate experiments (*n* = 3), with statistical significance at ∗*p* < 0.05 and ∗∗*p* < 0.01 (One-way ANOVA test). Scale bar: 100 and 200 μm.

Macrophage immunopolarization was evaluated by bulk RNA sequencing (RNA-Seq), fluorescent activated cell sorting (FACS), immunocytochemical (ICC) staining, and real-time polymerase chain reaction (qRT-PCR). M2 macrophages have been shown to promote wound healing by accelerating angiogenesis and fibroblast proliferation [35]. The RAW 264.7 cells were cultured on the freshly 3D printed constructs for 24 h. The details results of the AGP hydrogel fabrication, characterization, and 3D printing performances are discussed in Figs. S1–S4. It is well known that monocyte/macrophage polarization is greatly influenced by the chemical composition, porosity, and surface wettability of biomaterial scaffolds during tissue healing and regeneration [36,37]. Several reports have also demonstrated that nanoparticle-impregnated 3D hydrogels promote M2 macrophage polarization during the wound healing process [38]. The immunopolarized macrophage-derived Exos also exhibited phenotype-dependent proteins or miRNAs that trigger host immunity and tissue regeneration [39]. Moreover, the PDA-functionalized hydrogels and surfaces have been shown to enhance the M2 polarization of RAW 264.7 cells via *Src* or *AKT*/*STAT*-mediated intracellular signaling. In this regard, we showed that the 3D-printed AGP hydrogel promoted macrophage polarization towards the M2 (anti-inflammatory) axis via collective signaling pathways and the production of anti-inflammatory factors (IL-4 and IL-10). Fig. 1(a) shows a schematic overview of the macrophage polarization, their characterization, Exo cultivation, and 3D bioprinting of Exo-laden skin grafts. RNA-Seq is a powerful tool for analyzing the transcriptomic profiles of a single population. As shown in Fig. 1(b), a total of 959 genes and 788 genes were found to be up- and down-regulated in the presence of PDA-containing hydrogel compared to the control groups. When compared individually, a pronounced change in the M1/M2 switching was observed in the control and AGP groups. As shown in the Venn diagrams, the AG-treated group showed 118 genes related to M2 (∼13.93 %) and 98 genes related to the M1 phenotype, with 635 genes co-expressed in both phenotypes compared to the control groups (Fig. 1(c)). Interestingly, the AGP-treated groups exhibited enhanced expression of M2 genes rather than M1 genes. The total M2/M1 gene counts in AGP-1, AGP-2, and AGP-3 were 337/214 (∼39.78 %), 447/399 (∼52.77 %), and 473/291 (∼55.84 %), respectively. Therefore, we assumed that PDA NSP insertion into the AG matrix significantly upregulated M2-related genes compared to M1 genes. To gain insight into the biological functions and signature differentially expressed genes (DEGs) involved in M2 polarization, we performed k-means clustering analysis using the iDEP93 bioinformatics tool, and the results are shown in Fig. 1(d) *k*-means clustering (mean kurtosis value 0.74, adjusted R^2^ = 0.9877) was performed by selecting total genes (1747) related to immune response and extracellular matrix (ECM) signaling, followed by biclustering analysis to understand the enriched gene sets responsible for immunomodulation. Notably, cluster C exhibited distinct biological functions compared to clusters A or B across all samples, suggesting that Cluster-C has more significant genes related to immunomodulation. Moreover, biclustering analysis revealed that approximately 500 (p = 5.4e-258), 689 (p = 9.5e-297), and 279 (p = 5.1e-234) genes were related to immune effector process, immune response, and cytokine production, respectively. It was enriched in the KEGG pathway when compared between cluster C/A. We also identified DEGs from cluster C that are principally involved in immunomodulation, angiogenesis, and ECM signaling.

The KEGG enrichment analysis of the DEGs in Cluster-C of AGP-3 *vs*. Control revealed an interesting immune-regulatory activities in biological process (BP), cellular component (CC), and molecular function (MF) annotations. The AGP-3 vs. Control group displayed highest enrichment and gene counts for ‘*positive regulation of cytokine production*,’ ‘*response to external stimuli*,’ and ‘*secretion of inflammatory factors*,’ in BP (Fig. S5(a)) while a higher enrichment for ‘*transmembrane activity*,’ ‘*MHC-protein complex*,’ ‘*tyrosinase activity*,’ ‘*IL*-*4 receptor binding*,’ ‘*cytokine binding*,’ and ‘*receptor ligand activity*,’ in CC and MF annotations (Figs. S5(b and c)). A high-throughput text-mining results suggest that the higher enrichment of MHC-protein complex and IL-4 receptor binding activity is associated with M2 macrophage polarization of RAW 264.7 cells. Besides, a GSEA analysis of Cluster-C DEGs (Fig. S5(d)) in AGP-3 *vs*. Control group also depicted a higher gene size and ranking for ‘membrane activity,’ (MSigDB rank: 2, size: 58, NES: 1.922431) ‘response to polyamine,’ (MSigDB rank: 3, size: 10, NES: 1.536834) ‘JAK/STAT signaling,’ (MSigDB rank: 1, size: 37, NES: 1.567735) and ‘IL signaling’ (MSigDB rank: 2, size: 49, NES: 1.305067) suggesting the anti-inflammatory activation of RAW 264.7 cells in respond to AGP-3 scaffold. A STRING protein-protein interaction map of key DEGs in Cluster-3 is shown in Fig. S5(e). Based on the bioinformatics study, we recognized the M2 polarization of RAW 264.7 cells in the presence of AGP-3 scaffold and further examined the DEGs expression associated with cytokines, chemokines, angiogenic factors, and signaling molecules. As shown in Fig. 1(e), a dramatic increase in anti-inflammatory and decrease in pro-inflammatory cytokine-related genes was observed when the AGP-3/control samples were compared. The major upregulated anti-inflammatory genes included *IL-10* (2.0-fold), *IL-4* (>3.0 fold), *TGF-β* (>3.0 fold), *VEGF-A* (>10.0 fold), and *CSF-1* (>3.0 fold), respectively. Similarly, the major downregulated pro-inflammatory genes included *NOS2*/*iNOS* (<-1.0 fold), *IL-1β* (<-1.0 fold), *IL-6* (<-5.0 fold), *IL-ra* (<-2.0 fold), *IL-12* (<-1.0 fold), *IL-23* (<1.0 fold), and *TNF-α* (<-2.0 fold). Moreover, the major pro-inflammatory (downregulated; *CXCL9*, *CXCL10*, *CXCL11*, *CCL12*, and *CXCL7*) and anti-inflammatory (upregulated; *CXCL13*, *CXCL17*, *CCL13*, *CCL17*, *CCL22*, and *CCL28*) chemokines were also identified during immunomodulation of RAW 264.7 cells. M2 macrophages and macrophage-derived exosomes have also been shown to promote neo-angiogenesis during wound healing by upregulating the VEGF section from endothelial cells [40]. Based on this, we also investigated key regulatory pro-angiogenic genes during AGP-assisted immunopolarization. Also, among the top 12 pro-angiogenic genes, *HIF-1α* (3.5-fold) and *VEGF* (>10.0-fold) were significantly (Log2 fold, ∗*p* < 0.05) up-regulated in the presence of the AGP-3 hydrogel. Furthermore, ECM signaling-related genes, such as *JAK*, *STAT*, *SRC*, *MYD88*, *MERTK*, *PPARa*, and *PPARd*, and mechanosensitive genes, such as *TAZ*, were found to be significantly upregulated in the presence of the AGP-3 hydrogel compared to the control. It is well known that ECM signaling proteins play a considerable role in cell adhesion, proliferation, and differentiation. Our results demonstrated that the RAW 264.7 cells grown on AGP-3 scaffolds exhibited a distinct transcriptomic signature towards an anti-inflammatory phenotype, which motivated us to analyze the cytochemical profile of RAW 264.7 cells.

We further performed FACS and ICC to verify the bulk RNA-Seq data. It was interesting to note that the NOS2^+^ populations decreased after culturing onto the AGP scaffolds. Similarly, the percentage of CD163^+^ cells significantly increased in the AGP groups than in the AG group (Fig. 1(f)), which was in accordance with the RNA-Seq data. Moreover, the ICC analysis also showed similar results. As shown in Fig. 1(g), control macrophages exhibited higher expression of NOS2 (green), which was characterized by an intense fluorescence (FL) signal from the cytoplasm. The AGP-3 treated groups were also positive for NOS2, but the expression profile was relatively low compared to the control group. On the other hand, the expression of VEGF and CD163 was found to be significantly higher in the AGP-3 groups than in the control group, characterized by strong FL signals from the cytoplasm. It was also interesting to note that M1 macrophages exhibited a nearly oval-to-round morphology with giant nuclei.

Conversely, M2 macrophages showed a relatively small nucleus with elongated fibroblastic morphology, which is the hallmark of M2 polarization [40]. To verify immunomodulation and signaling pathways at the genetic level, we also examined the mRNA expression profile of signaling markers identified from RNA-Seq (*STATs*, *IL-4R*, and *RTK*), M1 polarization (*NOS2*, *TNF-α*, *IL-1β*, and *IL-6*), and M2 polarization (*CD163*, *VEGF*, *IL-4*, *IL-10*, *Arg-1*, and *TGF-β*) markers, respectively (Fig. 1(h)). The mRNA expression of *STAT3*, *IL-4R*, and *RTK* was found to be significantly (∗∗*p* < 0.01 and ∗∗∗*p* < 0.001) higher than the control, which supports the RNA-Seq data. Also, the expression of gene markers *VEGF*, *IL-4*, and *IL-10* was profoundly increased. By contrast, the expression of TNF-α and IL-1β was not significantly higher than that in the control, suggesting that M1 genes were significantly downregulated during macrophage differentiation. A schematic illustration of AGP scaffold-guided molecular activation of RAW 264.7 cell is demonstrated in Fig. S5(f). Taken together, these results suggested that the Group-I bioink made up of AGP would be highly immunomodulatory, which motivated us to cultivate the Exo from the M2 polarized macrophages (=M2-Exo) in further studies.

### 3.2 M2-Exo extraction and *in vitro* bioactivity evaluation

M2-Exo were extracted from M2 polarized macrophages grown on the AGP-3 scaffold (designated mExo-AGP) after 24 h of incubation. For control experiments, Exos (designated as mExo-Ctrl) were cultivated from AG scaffolds. Exos were characterized using nano-tracking analysis (NTA), transmission electron microscopy (TEM), and Western blotting (WB) analysis. The NTA analysis showed that the average hydrodynamic size of the mExo-Ctrl and mExo-AGP were 147 ± 2.8 nm and 179 ± 1.2 nm, respectively (Fig. 1(i)). We also found several particles ranging between 72 and 129 nm, which resembles the typical exosome size, as reported earlier [41,42]. Furthermore, the TEM results also showed that both Exos had a round-to-oval-shaped morphology with an average particle size of 60–140 nm, which is in accordance with the NTA data. To confirm the presence of Exos, we evaluated the expression of CD9 and CD63, which are specific to Exos; the results are shown in Fig. 1(j). Notably, both mExo-Ctrl and mExo-AGP displayed significant enrichment in CD9 and CD63 expression, suggesting that the extracted micro-vesicles are typical Exos [43].

To investigate exosome uptake and delivery efficiency, we labeled exosomes with 10 μL of Alexa Fluor-conjugated 488 probes (AF488, green)/100 μL of Exo. As shown in Fig. 1(k), mExo-AGP displayed a higher binding affinity towards AF488 dye than mExo-Ctrl, owing to the greater surface area and hydrodynamic size. The mExo-AGPs were then delivered into hDFs (10^8^ particles/100 μL) and visualized using a confocal laser microscope with an ex/em of 488/519 nm. At 0 h, no noticeable fluorescence was observed in the cells, except for the lysosomes, which stained red. After 1 h, a small bead-like fluorescent puncta was observed in the cytoplasm of the hDFs (Fig. S6). After approximately 2 h of incubation, several fluorescent puncta were observed in the cytoplasm, which were colocalized with lysosomes, indicating that the endocytosis of mExo-AGP was probably through the lysosomes. This result suggests the internalization pathway of mExo-AGP inside the cells.

Next, we examined the effects of Exos on skin and endothelial cell proliferation and migration potential after 24 h of incubation. The cytocompatibility of hDFs, hKCs, and hECs was evaluated using WST-8 assay. Notably, mExo-AGP significantly (∗*p* < 0.05) induced the proliferation of hDFs than the control and mExo-AG group (Fig. 1(l)). Similarly, hKCs and hECs showed superior cytocompatibility following mExo-AGP treatment, indicating the bioprotective role of M2-Exo (Fig. S7). We also observed an increase in hDF migration through the Transwell® chamber following Exos treatment. The percentage of migrated cells was significantly higher (∗∗*p* < 0.01) in mExo-AGP treated groups because of their biocompatible and immunity-boosting nature (Fig. S8). These results show that the Exos extracted from M2 polarized macrophages are highly biocompatible, non-toxic, and exhibit immune-boosting properties in skin and endothelial cells.

**Fig. 2:**
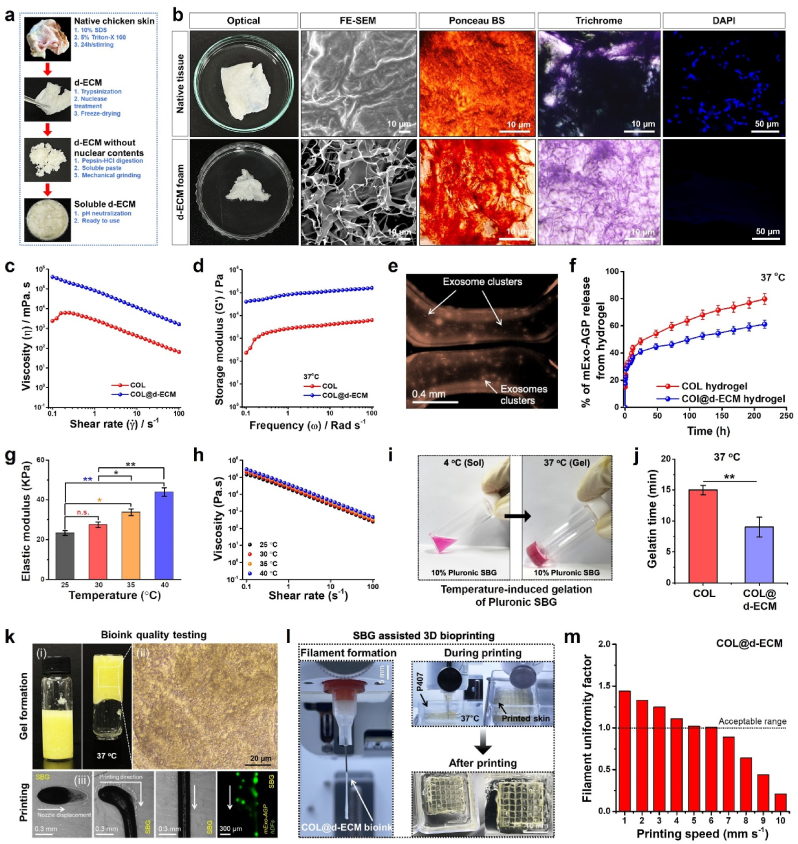
Fabrication of the d-ECM/exosome hydrogels and supporting bath (SBG)-assisted bioprinting. **(a)** Schematic diagram showing the overall extraction procedure of chicken skin d-ECM. **(b)** Morphological observation of the native skin and d-ECM mat (from left to right; digital photograph, FE-SEM, Ponceau BS, Trichrome, and DAPI staining). Scale bar: 10 and 50 μ m. **(c, d)** The flow curve and storage modulus of the pure COL and COL@d-ECM bioink. **(e)** Representative FL microscopy images of the 3D bioprinted COL@d-ECM hydrogel showing the localization of exosomes (mExo-AGP). Scale bar: 0.4 mm. **(f)** Evaluation of the exosome release from the COL@d-ECM hydrogel at indicated time points. **(g)** The elastic modulus of the 15 % (*w*/*v*) Pluronic SBG under varying temperatures. **(h)** The flow curve of the 15 % (*w*/*v*) Pluronic SBG under varying temperatures. **(i)** Digital photograph of the SBG showing the sol-gel transition in 4 °C/37 °C. **(j)** Representative gelatin time of the COL and COL@d-ECM hydrogels at 37 °C. Data reported are mean ± s.d. of triplicate experiments (*n* = 3), with statistical significance at ∗∗*p* < 0.01 (One-way ANOVA test). **(k)** Digital photograph of the gelation procedure of the COL@d-ECM bioink and in-bath 3D printing showing the exosome localization. Scale bar: 20, 300 μ m, and 0.3 mm. **(l)** COL@d-ECM bioink showing the uniform filament formation at 37 °C with outstanding printing ability. Scale bar: 5 and 10 mm. **(m)** Calculation of the embedded filament uniformity of COL@d-ECM as a function of printing speed at 37 °C.

Macrophage-derived exosomes have a positive role in inducing angiogenesis by boosting the secretion of VEGF and CSF-1 [[44], [45], [46], [47]]. To investigate the effect of Exos on angiogenesis, we performed a tube formation assay with and without Exo treatment (Fig. 2(m)). On day 1, hECs exhibited tube-like morphology and fused vessel structures in all groups. Interestingly, after three days, the mExo-AGP-treated samples exhibited higher tube formation efficiency than the mExo-Ctrl and negative control groups, suggesting that mExo-AGP treatment significantly affected the tube formation process. Moreover, we observed an enhancement in CD31 (a marker of angiogenesis) expression after 3 days of incubation. The number of vessels and total vessel area (Fig. 2(n)) were also higher in the presence of mExo-AGP. M2-polarized exosomes carry various proteins and miRNAs that can promote the angiogenesis of hECs via the paracrine signaling of VEGF, CSF, PDGF, and others [44]. Taken together, our results demonstrated that M2-Exos cultivated from AGP scaffolding platform plays a crucial role in cell proliferation and angiogenesis induction, which could be beneficial for skin-tissue engineering. At this point, we have selected only the mExo-AGP (=M2-Exo) for 3D bioprinting and biological studies and not the mExo-AG (=control Exo) based on the initial screening unless stated elsewhere.

**Fig. 3:**
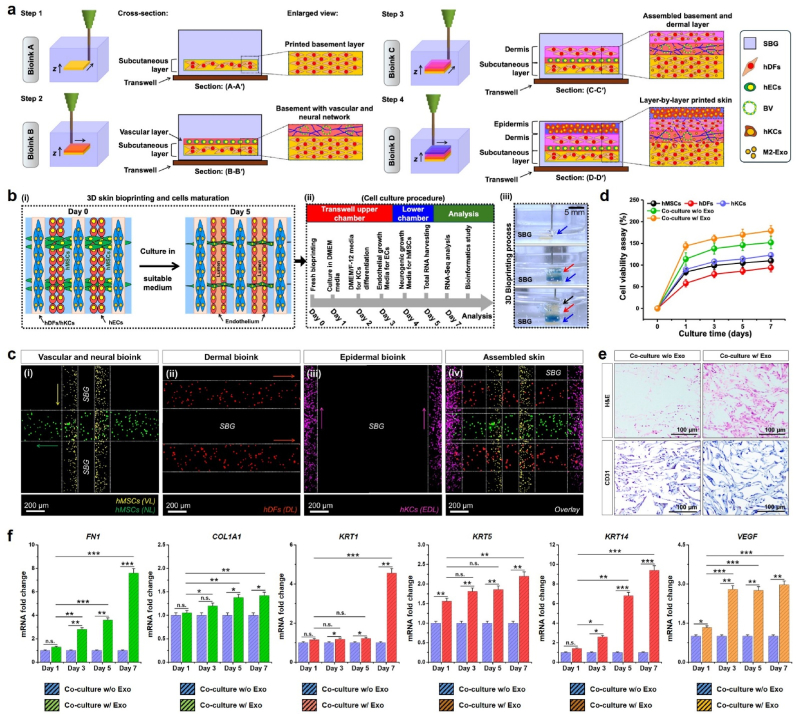
3D bioprinting of M2-Exo and skin cell-laden multilayered graft for wound healing study. **(a)** Schematic illustration of the SBG-assisted skin bioprinting procedure. **(b)** Schematic illustration of the 3D bioprinting and cell culture process used in this study. The bioprinting was conducted using epidermal, dermal, vascular, and neural bioinks (i&iii). The skin graft was supplemented with cell-specific media and incubated for 7 days prior to analysis (ii). Color hydrogel is shown for eye guidance. Scale bar: 5 mm. **(c)** Representative cytotoxicity assay of the cells at indicated time points. **(d)** FL microscopy images of the cell-laden constructs with individual layer components. Scale bar: 200 μm. **(e)** Representation optical micrographs of the epidermal/dermal region of the skin constructs showing the presence of cells and expression of CD31 marker in the presence of absence of Exos. Scale bar: 100 μm. **(f)** Representative qRT-PCR analysis showing the expression of major dermal (*FN* and *COL*), epidermal (*KRT1*, *KRT5,* and *KRT14*), and endothelial (*VEGF*) gene markers expression in a bioprinted skin model w/or w/o mExo-AGP. Data reported here are mean ± s.d. of triplicate experiments (*n* = 3), statistically significant at ∗*p* < 0.05, ∗∗*p* < 0.01, and ∗∗∗*p* < 0.001 (n.s. = not significant).

### 3.3 Characterization of d-ECM and COL@d-ECM/M2-Exo hydrogels

Native chicken skin was decellularized to obtain a soluble d-ECM. Chicken skin was initially chosen because of its easy availability in the local market and cost-effectiveness. Decellularization usually involves the removal of internal cellular contents, such as DNA, RNA, and proteins, without damaging the ECM structure [33,34]. d-ECM-based bioinks are highly favorable for developing *in vitro* tissue constructs to study the tissue microenvironment and cellular homogeneity [48]. Furthermore, the internal cellular components of d-ECM may induce toxicity when transplanted *in vivo* and thus should be checked for cellular components less than 2–5% before bioink formulation [34]. Herein, we successfully converted native chicken skin into printable d-ECM paste. A schematic diagram of the decellularization process is shown in Fig. 2(a). Due to the physical, chemical, and enzymatic hydrolysis, we removed the cellular components and analyzed them using FE-SEM, Ponceau BS staining, trichrome staining, and DAPI staining, respectively. As shown in Fig. 2(b), the freeze-dried d-ECM foam appeared pale white in color compared to the fresh chicken skin. Moreover, the FE-SEM morphology of the native skin exhibited irregular, wavy, or corrugated lamina on the basement membrane, as reported previously [49]. In contrast, the d-ECM foam exhibited a reticulated and fibrous structure, suggesting that the internal tissue was completely removed during decellularization. Ponceau BS and Trichrome staining results showed that the d-ECM matrix was positive for ECM-specific proteins and collagen fibers. This was further confirmed using DAPI staining. Notably, the cross-sectional morphology of d-ECM exhibited no traces of nuclei, whereas the native skin displayed abundant nuclei in the ECM matrix. These results suggest the successful decellularization of the native tissue. Next, we evaluated the quantities of DNA, collagen, and glycosaminoglycans (GAGs) in both the native tissue and d-ECM matrix and the results are shown in Fig. S9. As shown in Fig. S9(a), we observed a significant decrease (∗∗*p* < 0.01) in DNA content in the d-ECM (∼0.8 %) compared to that in the native skin, which supports the decellularization process. Since natural ECM is composed of various proteins and glycoproteins, we also quantified the relative content of collagen and GAGs in both native and d-ECM matrices. We observed a slight decrease in collagen content (Fig. S9(b)) in the d-ECM compared to that in the native tissue, which was due to the acidolysis and hydrolysis of the native ECM [34]. However, the GAGs content was significantly (∗*p* < 0.05) lower in the native d-ECM than in the native tissue, suggesting a complete decellularization process (Fig. S9(c)). The mechanical properties of the ECM scaffold are crucial for successful biomedical applications [50,51]. To investigate the mechanical properties, we performed tensile measurements on both native skin and d-ECM tissue. As shown in Fig. S9(d), the native skin had an elastic modulus of *E* = 33.8 ± 2.5 kPa, while the d-ECM showed an elastic modulus of *E* = 18.7 ± 1.9 kPa, respectively. The significant decrease (∗*p* < 0.05) in elastic modulus was probably due to the decellularization process, which resulted in a porous and fibrillar network resembling previous literatures [49]. Taken together, our results demonstrate the successful extraction of d-ECM from chicken skin, rich in proteins and can be utilized as a bioink for 3D printing applications.

To enhance the mechanical property, bioprintablity, and biocompatibility, we incorporated the rat tail type-1 collagen (=COL) with d-ECM hydrogel and loaded with mExo-AGP. We also prepared COL hydrogel as control group. The viscoelasticity of the fabricated COL and COL@d-ECM hydrogels were examined by a rotation rheometer to examine its feasibility as a bioink. The hydrogels were measured under varying shear rates ($\dot{\gamma }$ = 0.1 to 100 s^−1^) at 37 °C with an angular frequency of 10 Rad s^−1^. As shown in Fig. 2(c), both the hydrogels exhibited shear-dependent viscosity changes within the measured range. At low shear rates ($\dot{\gamma }$ = 0.1), the viscosities of the COL and COL@d-ECM hydrogels were calculated as 357 and 598 mPa, respectively. s. As the shear rate increased from 0.1 to 100 s^−1^, we observed a drastic decrease in viscosity, which resembles the typical shear-thinning nature. During shear stress, the hydrogel network undergoes temporary deformation, which is characterized by the breaking of the polymeric network. As the shear was removed, the hydrogel tended to rejoin, and reformation occurred. The continuous breaking and rejoining of the polymer matrix at varying shear rates supports the ideal shear-thinning nature of the developed COL@d-ECM [[52], [53], [54]]. We also observed an enhancement of the storage modulus (G′) in the COL@d-ECM hydrogel (158854 Pa) compared to that in the pure COL hydrogel (6240 Pa) (Fig. 2(d)), suggesting that composite proteinaceous hydrogel has enhanced viscoelasticity than pure COL hydrogel.

It has been reported that controlled delivery of Exos from the hydrogel scaffold may promote tissue regeneration and boost angiogenesis during wound healing [39,55]. Thus, we encapsulated mExo-AGP in the hydrogel matrix and examined their release profile. Based on that precedent, we first labeled mExo-AGP with Dil and evaluated its fluorescence (FL) properties which is easy for tracking, followed by encapsulation into the COL and COL@d-ECM hydrogel (Fig. S10(a)). The FL image of the Dil-labeled Exo inside the hydrogel matrix is shown in Fig. 2(e). FL decay were investigated to ensure the stability of mExo-AGP@Dil in an aqueous medium. As shown in Fig. S10(b), the average FL of the pure mExo-AGP@Dil and mExo-AGP@Dil + COL@d-ECM were calculated to be 17.6 ± 0.6 ns and 12.8 ± 0.4 ns, respectively. The lower recovery rate of the hydrogel-encapsulated mExo-AGP@Dil was probably due to the losses in fluorescence from scattering through the hydrogel and the interaction of the dye with the ECM components. Next, we examined the Exo-release efficiency of the COL@d-ECM hydrogel at 37 °C. For the control experiment, pure COL hydrogel was used. The Exo release study was conducted over a period of 10 days, and the results are shown in Fig. 2(f). Interestingly, we observed an initial burst release of mExo-AGP within 2 days, followed by a controlled release up to 10 days from the COL@d-ECM hydrogel. On the other hand, the COL hydrogel exhibited a prolonged and higher degree of mExo-AGP release compared to the COL@d-ECM hydrogel, suggesting that the COL@d-ECM hydrogel is more suitable for the delivery of Exo in the initial wound repair and regeneration.

The superior Exo release capability of the COL@d-ECM hydrogel further motivated us to examine the *in vitro* biocompatibility of human cells. We investigated the biocompatibility of the COL@d-ECM hydrogel in terms of 3D tube formation assay (=angiogenesis) using hMSCs with or without Exos, which is pivotal and one of the major factors for subcutaneous wound healing. Matrigel-treated hMSCs were used as a control group. COL-treated hMSCs were used as a positive control. The COL@d-ECM and COL@d-ECM + mExo-AGP-treated hMSCs were used as experimental groups. All the groups exhibited higher rates of angiogenic sprout formation, except for the control group at 24 h time point (Fig. S11(a)). We observed a slight decrease in sprout formation in COL@d-ECM at 24 h time point. Notably, we observed a filament-like growth of angiogenic in the COL@d-ECM + mExo-AGP groups at 24 h time point. More interestingly, COL@d-ECM + mExo-AGP group significantly enhanced tube formation in terms of length and thickness compared to the other groups, indicating that a combination of d-ECM hydrogel with M2-Exo may trigger the angiogenesis in hMSCs. The semi-quantitative evaluation of bud's number, sprouts, tube height, and width are shown in Fig. S11(b). The expression of the CD31 gene marker, a key biomarker for angiogenesis was also found to be higher (∗∗*p* < 0.01) after 48 h of incubation in the COL@d-ECM + mExo-AGP-treated group than control, suggesting the 3D angiogenesis and superior biocompatibility of the COL@d-ECM + mExo-AGP hydrogel.

### 3.4 Bioprintablity assessment of COL@d-ECM/M2-Exo hydrogels

The outstanding biocompatibility and angiogenic ability of COL@d-ECM/M2-Exo further motivated the scrutiny of the bioprintablity properties. For 3D bioprinting, we used a supporting bath gel (SBG) composed of 10 % Pluronic (P407). 10 % SBG was chosen based on our previous reports [56,57]. An ideal SBG must have desirable gelation properties and self-healing features during 3D bioprinting to control the uniform filament formation [58,59]. To investigate the mechanical and viscoelastic properties, we first examined the behavior of SBG at varying temperatures. As shown in Fig. 2(g), the elastic modulus of the SBG increased significantly as the temperature increased from 25 °C to 40 °C, suggesting its temperature-induced micelle formation of the triblock copolymers [56]. Furthermore, we observed a temperature-dependent shear-thinning (Fig. 2(h)) of the SBG, ideal for printing applications. A digital photograph of the temperature-dependent SBG gelation procedure is shown in Fig. 2(i). We also tested the gelatin property of the COL and COL@d-ECM hydrogels before testing printability. Interestingly, both the COL and COL@d-ECM hydrogels exhibited superior gelation properties at 37 °C (Fig. 2(j)). The COL@d-ECM hydrogel displayed fast gelation than COL hydrogel owing to the rapid inter- and intra-molecular covalent and hydrogen bonding between α-chains and triple-helix junction zones of COL and/or d-ECM [60].

Next, we investigated the printing quality and filament formation to assess bioprintablity of the COL@d-ECM hydrogel. As shown in Fig. 2(k) (i), the composite bioink gelled at 37 °C after 9 min of incubation. Microscopic observations suggested that the COL@d-ECM hydrogel exhibited a dense fibrillar and granular network after gelation (Fig. 2(k) (ii)). Flow rate, extrusion pressure, and printing speed are important parameters for supporting bath-assisted 3D printing. Thus, filament formation is principally dependent on the printing speed (*v*_out_) and path speed (*v*_path_), which can be correlated with the Herschel-Bulkley flow model [61]. Thus, the velocity distribution (*v*_*z*_) at the end of the nozzle can be expressed as:

Eq. 1 $$v_{z}=\frac{\left(p_{0}-\sigma \right)+\rho gL}{4\eta_{0}L}\;\left(R^{2}-r^{2}\right)$$

where *p*_0_, *L*, *R,* and *r* are the inlet pressure, nozzle length, nozzle radius, and drop radius, respectively. Considering the bioink flow rate Q, the hydrogel extrusion velocity can be estimated by dividing the flow rate by the total nozzle exit area and is given by the following equation:

Eq. 2 $$v_{out}=\frac{\left(p_{0}-\sigma \right)+\rho gL}{8\eta_{0}L}\;R^{2}$$

Based on this, we estimated the effect of extrusion velocity on filament formation inside the P407 bath. We used a 250 μm metal-head nozzle for 3D bioprinting experiments. The higher printing speed limits filament formation owing to mixing with SBG and super-thinning of the bioink, which ultimately tends to break easily. We found a stable filament formation at *v*_*out*_ = 5.5 mm s^−1^, respectively. Digital photographs of the filament formation process as a function of printing speed are shown in Fig. 2(k) (iii). The FL image represents the hydrogel filament with M2-Exo (stained yellow) in clusters during filament formation test (Fig. 2(k) (iv)).

Mathematical modeling further motivated us to examine acellular printing ability using the SBG. As shown in Fig. 2l (right), the COL@d-ECM ink exhibited stable filament formation when extruding from a 37 ± 2 °C-controlled print-head. A digital photograph of the SBG-assisted 3D bioprinting process is shown in Fig. 2(i) (left), which demonstrates the excellent printability of the developed bioinks. We also examined the relation between printing speed *vs*. filament uniformity experimentally. As shown in Fig. 2(m), a printing speed below 5 mm s^−1^ resulted in thick filaments while a speed above 10 mm s^−1^ resulted extremely thin filaments. Besides, a proper filament formation was observed at 5–6 mm s^−1^ with excellent printing structure, which support the theoretical data. Based on these observations we concluded that COL@d-ECM hydrogel is suitable for bioprinting and subsequently used for encapsulating the skin cells for next experiments.

### 3.5 3D bioprinting of multilayered skin with COL@d-ECM/M2-Exo bioinks

The aforementioned bioink was used for printing multi-cell-laden skin grafts *in vitro*. Prior to bioprinting, hDFs, hKCs, and hMSCs were mixed with COL@d-ECM pre-gel solution. The bioinks were designated epidermal bioink (hydrogel + hKCs), dermal bioink (hydrogel + hDFs), lumen bioink (hydrogel + hMSCs), and neural bioink (hydrogel + hMSCs). Fig. 3(a) depicts an overview of the 3D bioprinting of full-thickness skin grafts using multi-cell-laden COL@d-ECM hydrogel ink. All the bioinks were carefully loaded onto the printing cartridge and incubated for 30 min at 37 °C. Next, the bioinks were loaded onto the bioprinter, and each layer was carefully printed within the SBG. The proposed structure of the printable construct is schematically shown in Fig. 3(b) (i). After bioprinting, the printed skin construct was carefully collected from the SBG and rinsed twice with ice-cold water to remove any traces of SBG. Next, the skin construct was incubated in DMEM for 24 h to ensure maximum cell growth. An overview of the cell culture procedure and differentiation method is shown in Fig. 3(b) (ii). Digital photographs of the bioprinting process are shown in Fig. 3(b) (iii). The bioinks were mixed with either rhodamine B (0.01 % in PBS) or Trypan blue (1 × in PBS) dye to visualize the layer-by-layer printing process.

Next, we evaluated cell homogeneity in each layer of the skin construct. As shown in Fig. 3(c) (i), the vascular and neural layers were printed first, followed by the dermal layer (Fig. 3(c)(ii)) and epidermal layer (Fig. 3(c) (iii, iv)). We observed a homogenous distribution of cells inside the bioink, suggesting high-resolution bioprinting. After the desired time period, the constructs were incubated with the WST-8 dye to assess viability. As demonstrated in Fig. 3(d), all cells remained viable for up to 7 days of culture in various media. Notably, the co-cultured skin graft supplemented with mExo-AGP exhibited a significant increase in viability compared with the individual culture groups, suggesting that mExo-AGP treatment significantly increased the proliferation and differentiation potential of the cultured cells. To examine tissue formation ability, we studied the histology of the epidermis/dermis region of the skin graft after 7 days of culture (Fig. 3(e)). Interestingly, H&E staining results showed that the co-culture model without Exo had less infiltration inside the hydrogel matrix. However, the Exo-treated model showed very good cellular infiltration and tissue-like morphology inside the hydrogel scaffold. This was further confirmed by immunohistological staining for CD31 (angiogenic marker). Notably, both culture models were positive for CD31 marker, which demonstrated that hMSCs with hDFs/hKCs together contributed to angiogenesis. Although there was no significant difference in the quantitative expression of CD31, the Exo-treated samples displayed better tissue-mimicking structures than the Exo-untreated samples, where cells were randomly distributed onto the hydrogel matrix.

Fibroblasts plays pivotal role during wound healing via secreting adhesive proteins, such as fibronectin (FN) and collagen-1A (COL1A) and contribute to re-epithelialization [62,63]. The migratory fibroblast also secretes various growth factors which induces the epithelial-mesenchymal transition in keratinocytes during wound remodeling via inducing an ECM protein, keratin (KRT) [64]. It has been reported that M2-polarized macrophages and their secreted exosomes carry several micro RNAs (miRNAs), which can modulate the keratinocyte migration towards wound bed and participates in epidermis formation and boost angiogenesis [47,65]. In this context, we aimed to investigate the expression profile of the key epidermal, dermal, and angiogenic gene markers using qRT-PCR to study the effect of bioprinted M2-Exo on skin regeneration. The results are shown in Fig. 3(f). Notably, a drastic change in mRNA expression was found in monoculture and co-culture groups w/or w/o Exo treatment. The expression of *FN* and *COL1A1* was significantly enhanced (∗∗*p* < 0.01 and ∗∗∗*p* < 0.001) at day 7 in co-culture w/Exo group than co-culture w/o Exo groups, suggesting that Exo application and co-culture might favor the fibroblast maturation and differentiation. Similarly, the expression of *KRT1*, *KRT5*, and *KRT14* were significantly enhanced (∗∗∗*p* < 0.001) in the co-culture w/o Exo group compared to the co-culture w/o Exo groups, indicating the role of Exos in keratinocyte differentiation. However, the expression of *VEGF* was not significantly changed after 3 days in the co-culture w/Exo group, meaning that the expression of *VEGF* in stem cells was maintained in a steady state for up to 7 days.

### 3.6 Bulk RNA-Seq identifies diverse regulatory pathways in bioprinted skin graft following M2-Exo treatment

**Fig. 4:**
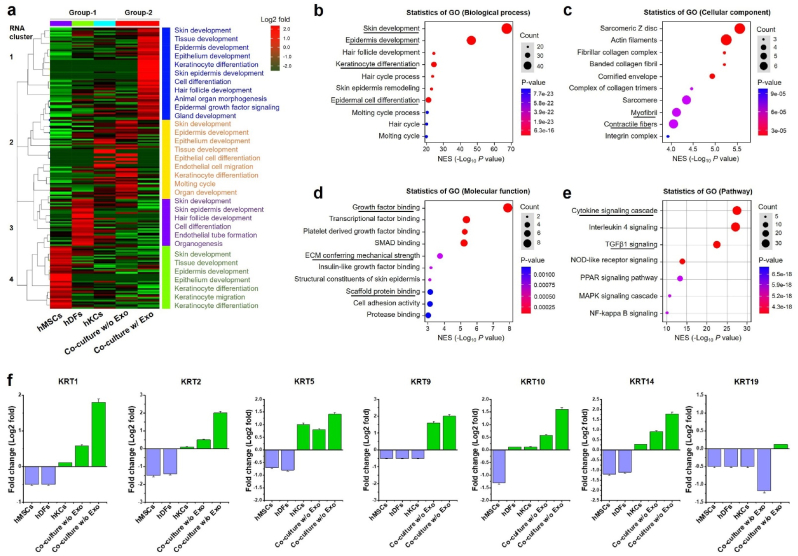
Transcriptomic changes of the bioprinted skin model w/or w/o exosomes. **(a)***k*-means RNA clustering of differentially expressed genes (DEGs) obtained from bulk RNA-seq related to skin development among the various groups after 7 days of *in vitro* culture. **(b**–**d)** The Gene set enrichment pathway analysis (GSEA) of co-culture w/Exo *vs*. co-culture w/o Exo with corresponding **(e)** gene ontology (GO) summary showing the major changes in biological process, cellular component, and molecular function, respectively. **(f)** Representative DEGs in various treatment groups showing the major changes in epidermal marker (KRT) expression w/or w/o Exo within the bioprinted COL@d-ECM hydrogel. Data represents the normalized RNA expression (Log2 fold, ∗*p* < 0.05 in various groups.

We examined the transcriptomic changes of the bioprinted skin model w/or w/o M2-Exo to understand the molecular basis and crosstalk between heterogeneous cells. Fig. 4(a) shows unbiased hierarchical clustering of the DEGs associated with skin tissue development in various groups. The individual (monoculture) bioprinted groups (hMSCs, hDFs, and hKCs group) exhibited various DEGs associated with skin development. Cluster-1 showed nearly similar type of DEGs expression related to skin/epidermis development for individual culture groups. Notably, we spotted a significant shift in the co-culture groups when treated w/Exos. The co-culture group w/o Exo showed a significant enhancement (average clustering co-efficient: 0.389; FDR: ∗*p* < 0.05) in epidermal gene markers than the co-culture w/o Exo group, respectively. The top epidermal markers enriched in cluster #1 of the co-culture w/Exo group includes late cornified envelope (*LEC1A*, *LEC2A*, and *LEC1H*), keratin (*KRT1*, *KRT2*, *KRT5*, *KRT9*, *KRT10*, *KRT14*, *KRT19, KRT75, KRT77,* and *KRT82*), insulin-like growth factor receptor (*IGFBP5*), arachidonate lipoxygenase 3 (*ALOX3*), corniodesmoitin (*CDSN*), sciellin (*SCEL*), transglutaminase-1 (*TGM1*), GATA binding factor-6 (GATA6), and leucine-rich G-protein coupled receptor-5 (*LGR5*). These genes were previously identified as putative markers for keratinocyte-fibroblast differentiation during skin re-epithelialization or wound healing [66,67]. Similarly, the fibroblast and endothelial gene signatures, such as fibronectin (*FN*) and collagen (*COL1A1, COL1A2, COL3A1, and COL5A2*), and vascular endothelial growth factor A (*VEGFA*) were also enriched (FDR ∗*p* < 0.05) in the co-culture w/o Exo group, compared to the co-culture w/o Exo group. These findings reflected that the bioprinted co-culture model w/Exo may have the capability to recapitulate the human skin. To claim this, we further studied the gene ontology (GO) of Cluster #1 of co-culture w/Exo group to find the significant function in biological process (BP), cellular component (CP), molecular function (MF), and GO pathways. As shown in Fig. 4(b-d), the above-mentioned genes were mostly co-related with major biological processes, such as ‘*skin development*,’ ‘*epidermis development*,’ ‘*keratinocyte differentiation*,’ and ‘*epidermal cell differentiation*’ related terms with a count of 30–40 % (∗*p* < 0.05). Similarly, the maximum hits were found for ‘*leucine-rich protein binding domains*’, ‘*collagen maturation*’, and ‘*contractile fibers*’ with a count value of 40 % in cellular components. Most of the molecular terms found in GO were related to either ‘*growth factor binding*’ or ‘*cell adhesion/scaffold binding*’ terms, significantly enriched (∗*p* < 0.05) with a count value > 40 %. A summary of various GO terms with corresponding enrichment scores was graphically shown in Fig. 4(e). We also examined the expression of various KRT gene families from bulk RNA-Seq data of Cluster-1 to find a correlation and co-expression pattern in the bioprinted skin grafts since ‘*keratinocyte differentiation*,’ and ‘*epidermal cell differentiation*’ were found a highly enriched term in Cluster-1. As shown in Fig. 4(f), the *KRT* gene family was the most highly expressed gene in the co-culture groups than the monoculture groups. The expression of *KRT* was significantly downregulated in monoculture groups, except the hKCs group.

Since epidermis development was found to be a highly significant (∗*p* < 0.05) term in the co-culture w/Exo group, we next evaluated the network profile of epidermal DEGs from the bulk RNA-Seq data and its relation to various signaling pathways using the STRING database and Cnet plot/GSEA. Interestingly, the expression of the focal adhesion gene (*YAP1*) and various signaling pathway genes (*SMAD4*, *PDGFA*, and *TGFB2*) was strongly upregulated in the co-culture groups while downregulated in the monoculture groups (Fig. S12(a)). The STRING text-mining and data-mining results showed that these genes were connected to the SMAD, platelet-derived growth factor (PDGF), fibroblast growth factor (FGF), and transforming growth factor β (TGFβ) signaling pathways during skin development. A visualized enrichment-based functional network is shown in Fig. S12(b). The GSEA results showed that most of the previously mentioned genes were significantly associated with epidermal and ECM receptor signaling pathways with an enrichment value of 30–40 %. The Cnet cord was found to be highly interconnected with the genes related to ‘epidermis development,’ ‘hair follicle development,’ and ‘keratinocyte differentiation,’ respectively (Fig. S12(c)). The Cnet cord was also enriched for signaling pathways related to ‘*ECM receptor*’ (cord size: 9; Log2 enrichment score: 2.0), ‘*focal adhesion*’ (cord size: 9; Log2 enrichment score: 1.8), ‘*mitogen-activated protein kinase (MAPK)*’ (cord size: 9; Log2 enrichment score: 1.0), and ‘*PI3k-Akt’* pathways (cord size: 9; Log2 enrichment score: 1.8) (Fig. S12(d)). Taken together, our results demonstrated that the M2 Exos had a potential role in skin development in terms of keratinocyte migration and differentiation towards epidermis remodeling.

### 3.7 *In vivo* wound regenerative potential

Biosafety is one of the major factors for successful *in vivo* application of any hydrogel scaffolds [68,69]. The *in vivo* biocompatibility of the 3D-printed hydrogels was evaluated through a hemolysis assay. Interestingly, the COL@d-ECM and COL@d-ECM + mExo-AGP hydrogels were found biocompatible with the RBCs of the rats, suggesting their blood biocompatibility (Fig. S13). Taken together, our results demonstrate that the COL@d-ECM bioink + mExo-AGP has excellent biocompatibility and the ability to form a skin-like structure that could be used for skin tissue engineering, especially for skin injury treatment.

**Fig. 5:**
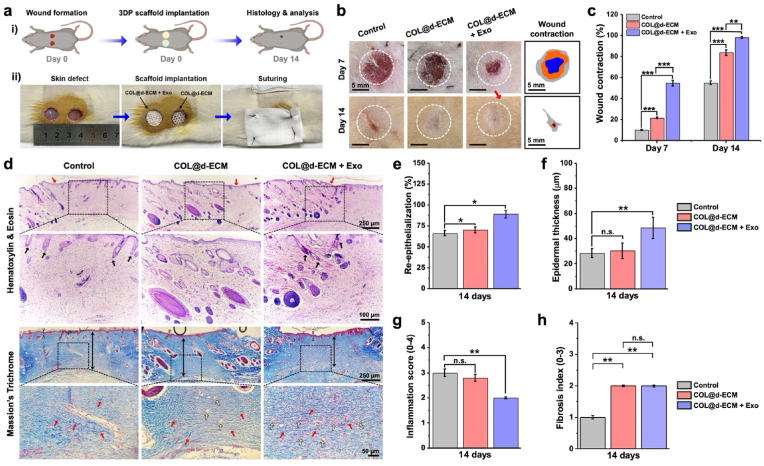
*In vivo* evaluation of wound healing in the presence of exosome-laden 3D bioprinted hydrogels. **(a)** Schematic illustration of the wound healing experiment **(i)** with corresponding surgical images **(ii)** showing the subcutaneous wound formation and scaffold implantation. Scale bar: 10 mm. **(b)** Digital images of the macroscopic wound healing study with corresponding wound contraction map. Scale bar: 5 mm. **(c)** Quantification data of the wound contraction rate (%) after 7 and 14 days of scaffold treatment. Data are mean +s.d. (*n* = 3), statistical significance at ∗∗*p* < 0.01 and ∗∗∗*p* < 0.001 (One-way ANOVA test). **(d)** Representative hematoxylin & eosin (H&E) and Massion's Trichrome staining images showing the microscopic wound healing. Red and black arrows in H&E staining indicate the presence of epidermis and hair buds/follicles; White triangle, red, and white arrows in the Trichrome staining indicate the epidermis, collagen fibrils, and blood vessels. Scale bar: 50, 100, and 250 μm. **(e**–**h)** Quantitative analysis (*n* = 5) of the *in vivo* wound healing; **(e)** Calculation of skin re-epithelialization, **(f)** epidermal thickness, **(g)** Inflammatory cells score (0–4 scale), and **(h)** fibrosis (scar) index. Data are mean +s.d. of five (*n* = 5) independent histological images, with statistical significance at ∗*p* < 0.05 and ∗∗*p* < 0.01 (One-way ANOVA test).

The *in vivo* wound regeneration potential of the COL@d-ECM + Exo scaffold was assessed in a rat subcutaneous wound model after 14 days of implantation. With that, COL@d-ECM scaffold (w/o Exo) was also tested as positive control, and wounds devoid of any scaffold were taken as negative control. Previous studies demonstrated that hADSCs-derived Exo encapsulated in chitosan hydrogel exhibited superior *in vivo* wound healing properties in diabetic wound models via inducing angiogenesis and collagen deposition [46,70]. In another study, Exo-laden cryogels have been shown to promote diabetic and infectious wound regeneration within 21 days of implantation [71]. Consistent with these reports, our study revealed fast and full-thickness subcutaneous wound healing upon Exo treatment. Fig. 5(a) shows the surgical procedure and outline of the wound healing study. After scaffold implantation, the macroscopic wound healing was photographed after 7- and 14-day intervals. As shown in Fig. 5(b), the general observation of the macroscopic wounds suggested the healing efficiency in all the groups, and the COL@d-ECM + Exo group showed a significant decrease in wound size compared to the control group after 14 days of incubation. The wound contraction rate (%) in various groups after 7 and 14 days of treatment is shown in Fig. 5(c). In the early healing stage (day 7), the COL@d-ECM and COL@d-ECM + Exo groups showed greater wound contraction rate than the control group, and the COL@d-ECM + Exo-treated group showed a significant increase (∗∗∗*p* < 0.001) in the wound contraction. However, at a later stage of healing (day 14), the control group showed scar formation, which was subsequently reduced in COL@d-ECM and COL@d-ECM + Exo-treated groups. These results indicated that COL@d-ECM + Exo groups had better wound healing performance compared to COL@d-ECM and control due to the controlled release of Exo and the ability to reduce scar formation.

To understand wound healing at the microscopic level, we further examined the wound healing rate using H&E and Massion's Trichrome staining. Fig. 5(d) shows the representative staining images of the wound bed after 14 days of implantation. Although all the treatment groups showed good regenerative properties with granulation and skin re-epithelialization, the COL@d-ECM + Exo-treated groups exhibited superior healing ability with thick epidermis, granulation tissue, various glands, and HF growth. Compared to the control group, the COL@d-ECM + Exo group demonstrated significantly (∗*p* < 0.05 and ∗∗*p* < 0.01) higher rate of skin re-epithelization and thick epidermis formation as depicted through H&E staining (Fig. 5(e and f)). The epidermis thickness in control, COL@d-ECM, and COL@d-ECM + Exo groups were calculated to be 28.43 ± 3.69, 30.36 ± 6.14, and 48.58 ± 8.49 μm, respectively. Moreover, the inflammation score was also calculated from the H&E staining images to understand the infiltration of immune cells. As shown in Fig. 5(g), the control and COL@d-ECM group displayed a bit higher rate of inflammatory cells with no significant differences. Besides, the COL@d-ECM + Exo scaffold treatment showed a reduction (∗∗*p* < 0.01) in inflammation score, suggesting that Exo therapy using an ECM mimicking hydrogel had the potential to reduce skin inflammation and thereby accelerate the wound healing process. These results were consistent with the qRT-PCR data of inflammatory gene markers analyzed from the 14-day wound bed. Interestingly, the expression of *IL-6* and *TNF-α* were gradually decreased in COL@d-ECM and COL@d-ECM + Exo-treated groups than control (Fig. S14), suggesting the attenuation of pro-inflammatory environments in the wound bed at day 14.

At the later stage of wound healing, fibroblast cells migrate toward the wound bed and contribute to the dermis formation. The fibroblast cells secrete collagen and contribute to the ECM formation [63]. Since d-ECM-based hydrogels contain various natural ECM proteins, including collagen, they are expected to heal skin wounds better than conventional hydrogels [72]. To evaluate the amount of collagen matrix deposition and fibrosis index (scar forming index) in the control and treatment groups, we further conducted the Massion's Trichrome staining and the results are shown in Fig. 5(h). The control group exhibited partial appearance of the granulation tissue (score: 1), while the wound bed of the COL@d-ECM and COL@d-ECM + Exo group displayed thin granulation tissue with moderate collagen deposition (score: 2), which could be due to the excessive activity of anti-inflammatory macrophages at or near the wound bed. It has been reported that during proliferation phase of wound healing, M2 macrophages induces the synthesis of ECM proteins, specifically collagen type-VIII which interacts with other ECM components and induces hypertrophic scar formation at around 21 days [73,74]. In this context, the fabricated COL@d-ECM + Exo hydrogel displayed mild fibrosis. In summary, our results demonstrated the therapeutic efficacy of the 3D printed COL@d-ECM + Exo hydrogels, where the sustained release of Exo contributed to the angiogenesis, epidermis remodeling, and collagen deposition, thereby making it an ideal implantable material for wound healing applications.

### 3.8 Robust immunomodulation and HF development at the scaffold-wound interface triggered by M2-Exo

**Fig. 6:**
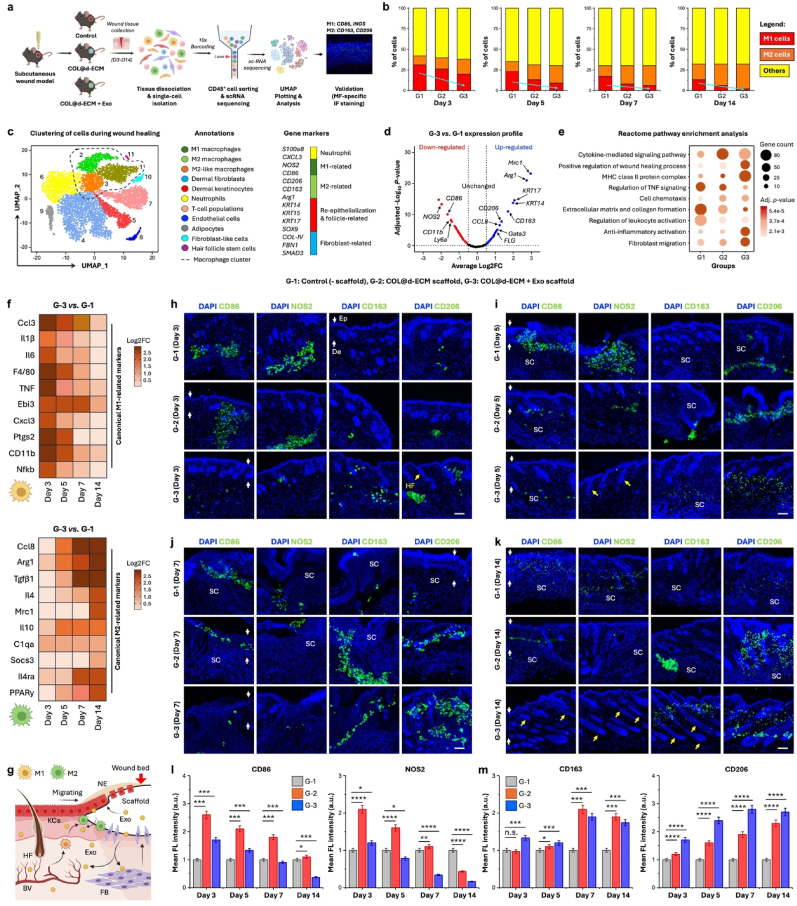
*In vivo* immunomodulatory property of the 3D bioprinted exosome-laden hydrogels for subcutaneous wound healing. **(a)** Schematic workflow of the immunomodulation study using scaffolds. **(b)** Percentage of the cells extracted from the wound tissue after *in vivo* immunomodulation study within a time-frame of 3–14 days post-implantation. The relative abundance of the macrophage (M1 and M2 phenotype) and other (dermal fibroblast, keratinocytes, neutrophils, T-cells, endothelial, adipocytes, and hair follicle stem cells) cells were analyzed from the single-cell atlas. The arrow indicates the change in M1 polarized macrophages within a time frame of 3–14 days. **(c)** scRNA sequencing analysis of the wound tissue. UMAP plot showing 11 different clusters and 5 subclusters with their annotations and marker genes. **(d)** Volcano plot showing the significantly up- and down-regulated genes in COL@d-ECM + Exo *vs*. Control (G-3 *vs*. G-1) after 14 days post-implantation. **(e)** Representative Reactome pathway enrichment analysis of the control (G-1), COL@d-ECM (G-2), and COL@d-ECM + Exo (G-3) groups after 14 days of implantation. **(f)** Differential gene expression of canonical M1 and M2 markers in the wound tissue within a time frame of 3–14 days. The heatmaps show the DEG expression associated with M1 and M2 macrophage polarization when compared between COL@d-ECM + Exo (G-3) and control (G-1). **(g)** Schematic illustration of biomaterial-assisted skin re-epithelialization and wound remodeling process through macrophage polarization. HF, hair follicle; KCs, keratinocytes; FB, fibroblast; BV, blood vessel; NE, neo-epidermis. **(h**–**k)** Representative immunofluorescence staining of wound tissues showing the expression of pro-inflammatory (CD86 and NOS2) and anti-inflammatory (CD163 and CD206) markers at indicated time points. Scale bar: 100 μ m. **(l, m)** The mean fluorescence intensity of various immunogenic markers. Data reported as mean +s.d. of triplicated (*n* = 3) experiments, statistical significance considered at ∗*p* < 0.05, ∗∗*p* < 0.01, ∗∗∗*p* < 0.001, and ∗∗∗∗*p* < 0.0001 (student *t*-test).

To explore the underlying mechanism of the immunomodulation-assisted wound healing process, we studied the time-dependent activation of the M2-macrophage polarization process and its role in HF development after implantation of COL@d-ECM + M2-Exo scaffold. Fig. 6(a) shows the schematic workflow of the analysis process. The time-dependent activation of various inflammatory markers was examined using scRNA-Seq to access the spatial distribution of canonical M1 and M2 polarization markers, followed by validation using immunocytochemistry. We isolated the wound tissue after 3, 5, 7, and 14 days of post-implantation and subjected them to scRNA-seq. Before the data processing, the raw data was undergone a quality control process, followed by analysis of the unique molecular identifier (UMI) profiles using *CellRanger* software (Figs. S15(a–d)). The three major cell populations were initially categorized as M1-polarized (*Ptgs2*^+^*CD86*^+^*Nos2*^+^), M2-polarized (*Mrc1*^+^Arg*1*^+^*C1qa*^+^), and others (epithelial, endothelial, and associated cells, *Pdgfra* ^+^ *Smad3*^+^ or *Krt5*^+^*Krt14*^+^ or *Gata3*^+^*Il4*^+^) after cell sorting and filtering. As shown in Fig. 6(b), the amount of pro-inflammatory or M1-cells gradually decreased in COL@d-ECM + Exo group (G-3) from day 3 to day 14 in the wound bed, compared to the control (G-1) and COL@d-ECM (G-2) groups, suggesting that the Exo-containing hydrogel had better anti-inflammatory properties. The Seurat unsupervised clustering revealed 11 main clusters during the subcutaneous wound healing process based on global gene expression profile. The uniform manifold approximation and projection (UMAP) clustering with corresponding annotation and marker genes during scaffold-assisted wound healing is documented in Fig. 6(c). The major gene markers in COL@d-ECM + M2-Exo groups that were differentially regulated were identified for neutrophil activation (*S100a8* and *CXCL3*), pro-inflammatory factors (*NOS2* and *CD86*), anti-inflammatory factors (*CD206*, *CD163*, and Arg*1*), skin re-epithelialization/HF development (*KRT14*, *KRT15*, *KRT17*, and *SOX9*), fibroblast migration (*COL-IV*, *FBN1*, and *SMAD3*) from clusters 6, 1, 2, 5, and 4 at day 14. Moreover, the volcano plot showed at least 20 key genes associated with M1-macrophage polarization were significantly (Log2FC, ∗*p* < 0.05) down-regulated (Fig. 6(d)) during the wound-healing process when compared between COL@d-ECM + Exo and control groups. We observed a down-regulation of signature pro-inflammatory markers (*CD86*, *NOS2*, and *CD11b*) at day 14 in the COL@d-ECM + M2-Exo group. Besides, in accordance with other reports, the expression of keratin 14 and 17 (*KRT14* and *KRT17*), the putative markers of HF development, were found to be highly up-regulated in COL@d-ECM + Exo-primed wounds, suggesting the intermediate stage of wound healing [75]. We also observed that GATA binding protein 3 (*Gata3*), a signature gene marker for both M2-macrophage (Arg*1*^+^*Gata3*^+^) and T-regulatory (Tregs; *Gata3*^+^*Il4*^+^ or *CD3*^+^) cells associated with Th2-mediated immune response and collagen deposition, was significantly up-regulated in COL@d-ECM + M2-*Exo*-treated group, further suggesting the role of adaptive immune response at or near the wound bed at day 14 [76,77].

The ‘Reactome’ pathway enrichment analysis (Fig. 6(e)) reveals that the genes highly up-regulated in COL@d-ECM + M2-*Exo*-primed wounds were correlated with higher gene counts for *cytokine-mediated signaling* (*p* = 5.1e-5, gene count = 80), *positive regulation of wound healing process* (*p* = 5.3e-5, gene count = 50), *MHC class II protein complex* (*p* = 5.8e-5, gene count = 80), and *anti-inflammatory activation* (*p* = 5.0e-5, gene count = 52), while negatively correlated with less gene counts for *TNF signaling* (*p* = 2.3e-3, gene count = 10) and *ECM and collagen formation* (*p* = 5.6e-5, gene count = 11) at day 14. The scRNA expression of canonical (=classical) M1 and M2 markers from the wound tissue suggested a gradual activation of anti-inflammatory markers and cytokines (Fig. 6(f)) when compared between COL@d-ECM + M2-Exo *vs*. the control group. For instance, the expression of *Ccl3*, *Il1β*, *Il6*, *TNF*, *Ptgs2*, *Ebi3*, *CD11b*, and *Nfkb* were down-regulated while the expression of *Ccl8*, Arg*1*, *Tgfβ1*, *Il10*, *Mrc1*, *Il4ra*, and *PPARy* were found up-regulated in COL@d-ECM + M2-*Exo*-treated wounds in 3–14 days. It has been reported that *Il4ra* plays an important role in M2-macrophage polarization and orchestrates wound healing by inducing collagen deposition [78]. Taken together, the scRNA-Seq analysis of the wound tissue suggested the robust anti-inflammatory activation of macrophages, scar contraction, and hair follicle development triggered by COL@d-ECM + M2-Exo scaffolds. A schematic illustration of the biomaterial-assisted wound healing predicted through scRNA-Seq data is given in Fig. 6(g).

To validate the immunomodulatory effects of the exosome-laden hydrogel, the wound tissue was subjected to cytochemical staining against CD86, NOS2, CD163, and CD206 after 3, 5, 7, and 14 days postoperatively. Previous studies reported that skin-derived d-ECM hydrogels can trigger *in vivo* wound healing by inducing the activity of anti-inflammatory factors after 14–28 days of treatment [79,80]. Immunofluorescence staining results showed a gradual increase in CD86^+^ and NOS2^+^ cells in control and COL@d-ECM groups after 3 days’ post-implantation (Fig. 6(h)). However, the COL@d-ECM + M2-Exo group displayed reduced expression of CD86^+^ and NOS2^+^ cells, followed by a significant (∗∗∗*p* < 0.001) increase in CDD163^+^ and CD206^+^ cells. Surprisingly, the expression of CD163^+^ and CD206^+^ cells were more predominant at day 5, 7, and 14 in COL@d-ECM + Exo group than other groups (Fig. 6(i–k)), suggesting that M2-Exo played a positive role in wound healing by accumulating the anti-inflammatory cells and their secreted cytokines at or near the wound bed, which later mobilizes the fibroblast differentiation and eventually activates the keratinocytes towards neo-epidermis development. The quantitative analysis of macrophage polarization is shown in Fig. 6(l). With that, the immunostaining images also suggest a significant amount of HF development in COL@d-ECM + M2-Exo groups at day 14 compared to other groups, further suggesting the robust wound remodeling potential of the bioprinted hydrogels.

## 4 Conclusion

In summary, the fabricated COL@d-ECM/M2-Exo (=mExo-AGP) hydrogel exhibited a significant potential in skin re-epithelialization and subsequent wound healing. The immunopolarized exosomes generated from a catecholamine-modified biomaterial platform (AGP scaffold) showed excellent bioactivity towards human skin cells and accelerated 3D angiogenesis. In-depth morphological and functional analysis revealed the involvement of JAK/STAT, IL-4R, and RTK signaling pathways involved in M2 macrophage polarization onto the AGP substrate. The shear-thinning COL@d-ECM + M2-Exo hydrogel was found highly biocompatible towards hDFs, hECs, and hKCs, and the sustained release of M2-Exo from the bioprinted hydrogel allowed a robust wound healing at day 14 by inducing thick epidermis formation, reducing pro-inflammatory activity, and HF induction. In a co-culture *in vitro* bioprinting model, the M2-Exo present in COL@d-ECM hydrogel promoted the hDFs maturation and collagen deposition and showed enhanced cytokeratin (*KRTs*) secretion by the hKCs. Moreover, the qRT-PCR and RNA-Seq study revealed that M2-Exo specifically activated the pathways associated with epidermal growth factor signaling and SMAD signaling during wound healing. Furthermore, we elucidated the underlying molecular mechanisms of the *in vivo* immunomodulation and wound-healing properties of the COL@d-ECM/M2-Exo hydrogel, revealing the activation of key anti-inflammatory factors (CD163 and CD206) throughout the healing process. We observed good blood biocompatibility of COL@d-ECM + M2-Exo *in vivo*, suggesting their biosafety and long-term therapeutic potential. Looking forward, we anticipate that M2-Exo/skin cells-loaded printable inks will present many opportunities in skin tissue engineering. Our findings suggest that a smart bioprinting platform consisting of ECM polymers, M2-Exo, and skin cells will mimic the naïve skin and offer a new therapeutic strategy for developing personalized skin grafts, especially for traumatic wound healing.

## CRediT authorship contribution statement

**Sayan Deb Dutta:** Writing – review & editing, Writing – original draft, Visualization, Supervision, Project administration, Methodology, Investigation, Data curation, Conceptualization. **Jeong Man An:** Formal analysis, Methodology. **Jin Hexiu:** Methodology, Data curation. **Aayushi Randhawa:** Software, Methodology, Formal analysis, Data curation. **Keya Ganguly:** Writing – review & editing. **Tejal V. Patil:** Writing – review & editing. **Thavasyappan Thambi:** Writing – review & editing, Methodology, Data curation. **Jangho Kim:** Funding acquisition, Project administration. **Yong-kyu Lee:** Project administration, Supervision, Writing – review & editing. **Ki-Taek Lim:** Writing – review & editing, Supervision, Resources, Investigation, Funding acquisition.

## Data availability

The raw data required to support this finding can be obtained from the corresponding author upon request.

## Ethics approval and consent to participate

All the animal experiments were approved by the Capital Medical University Animal Experimental Ethics Committee (Permission No: KQYY-202012-004), Beijing, China. The experiments were performed in a blinded fashion.

## Declaration of competing interest

The authors declare no completing financial interests.

## References

[1] Landén N.X., Li D., Ståhle M. (2016). Transition from inflammation to proliferation: a critical step during wound healing. Cell. Mol. Life Sci..

[2] Veith A.P., Henderson K., Spencer A., Sligar A.D., Baker A.B. (2019). Therapeutic strategies for enhancing angiogenesis in wound healing. Adv. Drug Deliv. Rev..

[3] Xie Y., Yu L., Cheng Z., Peng Y., Cao Z., Chen B. (2022). SHED-derived exosomes promote LPS-induced wound healing with less itching by stimulating macrophage autophagy. J. Nanobiotechnol..

[4] Zhang B., Wang M., Gong A., Zhang X., Wu X., Zhu Y. (2015). HucMSC-exosome mediated-Wnt4 signaling is required for cutaneous wound healing. Stem Cell..

[5] Li J., Jiang X., Li H., Gelinsky M., Gu Z. (2021). Tailoring materials for modulation of macrophage fate. Adv. Mater..

[6] Wynn T.A., Barron L. (2010). Seminars in Liver Disease.

[7] Murray P.J., Wynn T.A. (2011). Protective and pathogenic functions of macrophage subsets. Nat. Rev. Immunol..

[8] Xiong Y., Mi B.-B., Lin Z., Hu Y.-Q., Yu L., Zha K.-K. (2022). The role of the immune microenvironment in bone, cartilage, and soft tissue regeneration: from mechanism to therapeutic opportunity. Mil. Med. Res..

[9] Patel D.K., Dutta S.D., Hexiu J., Ganguly K., Lim K.-T. (2022). 3D-printable chitosan/silk fibroin/cellulose nanoparticle scaffolds for bone regeneration via M2 macrophage polarization. Carbohydr. Polym..

[10] Dutta S.D., Ganguly K., Patil T.V., Randhawa A., Lim K.-T. (2023). Unraveling the potential of 3D bioprinted immunomodulatory materials for regulating macrophage polarization: state-of-the-art in bone and associated tissue regeneration. Bioact. Mater..

[11] Guan Y., Racioppi L., Gerecht S. (2023). Engineering biomaterials to tailor the microenvironment for macrophage–endothelium interactions. Nat. Rev. Mater..

[12] Fuchs A.-K., Syrovets T., Haas K.A., Loos C., Musyanovych A., Mailänder V. (2016). Carboxyl-and amino-functionalized polystyrene nanoparticles differentially affect the polarization profile of M1 and M2 macrophage subsets. Biomaterials.

[13] Yang Y., Guo L., Wang Z., Liu P., Liu X., Ding J. (2021). Targeted silver nanoparticles for rheumatoid arthritis therapy via macrophage apoptosis and Re-polarization. Biomaterials.

[14] Wu J., Zhu J., Wu Q., An Y., Wang K., Xuan T. (2021). Mussel-inspired surface immobilization of heparin on magnetic nanoparticles for enhanced wound repair via sustained release of a growth factor and M2 macrophage polarization. ACS Appl. Mater. Interfaces.

[15] Xiong Y., Feng Q., Lu L., Qiu X., Knoedler S., Panayi A.C. (2024). Metal–organic frameworks and their composites for chronic wound healing: from bench to bedside. Adv. Mater..

[16] Gong J., Ye C., Ran J., Xiong X., Fang X., Zhou X. (2023). Polydopamine-mediated immunomodulatory patch for diabetic periodontal tissue regeneration assisted by metformin-ZIF system. ACS Nano.

[17] Xu J., Younis M.R., Zhang Z., Feng Y., Su L., Que Y. (2023). Mild heat-assisted polydopamine/alginate hydrogel containing low-dose nanoselenium for facilitating infected wound healing. ACS Appl. Mater. Interfaces.

[18] Wang Y., Xiao D., Quan L., Chai H., Sui X., Wang B. (2022). Mussel-inspired adhesive gelatin–polyacrylamide hydrogel wound dressing loaded with tetracycline hydrochloride to enhance complete skin regeneration. Soft Matter.

[19] Sheng W., Song Q., Su X., Lu Y., Bai Y., Ji F. (2023). Sodium alginate/gelatin hydrogels loaded with adipose‐derived mesenchymal stem cells promote wound healing in diabetic rats. J. Cosmet. Dermatol..

[20] Luo P., Huang R., Wu Y., Liu X., Shan Z., Gong L. (2023). Tailoring the multiscale mechanics of tunable decellularized extracellular matrix (dECM) for wound healing through immunomodulation. Bioact. Mater..

[21] Xiong Y., Lin Z., Bu P., Yu T., Endo Y., Zhou W. (2023). A whole‐course‐repair system based on neurogenesis‐angiogenesis crosstalk and macrophage reprogramming promotes diabetic wound healing. Adv. Mater..

[22] Norouzi-Barough L., Shirian S., Gorji A., Sadeghi M. (2022). Therapeutic potential of mesenchymal stem cell-derived exosomes as a cell-free therapy approach for the treatment of skin, bone, and cartilage defects. Connect. Tissue Res..

[23] Ma Y., Wang Y., Chen D., Su T., Chang Q., Huang W. (2023). 3D bioprinting of a gradient stiffened gelatin–alginate hydrogel with adipose-derived stem cells for full-thickness skin regeneration. J. Mater. Chem. B.

[24] Bian S., Hu X., Zhu H., Du W., Wang C., Wang L. (2024). 3D bioprinting of artificial skin substitute with improved mechanical property and regulated cell behavior through integrating patterned nanofibrous films. ACS Nano.

[25] Lihao Q., Tingting L., Jiawei Z., Yifei B., Zheyu T., Jingyan L. (2024). 3D bioprinting of Salvianolic acid B-sodium alginate-gelatin skin scaffolds promotes diabetic wound repair via antioxidant, anti-inflammatory, and proangiogenic effects. Biomed. Pharmacother..

[26] Chen S., Xiong Y., Yang F., Hu Y., Feng J., Zhou F. (2024). Approaches to scarless burn wound healing: application of 3D printed skin substitutes with dual properties of anti-infection and balancing wound hydration levels. EBioMedicine.

[27] Motter Catarino C., Cigaran Schuck D., Dechiario L., Karande P. (2023). Incorporation of hair follicles in 3D bioprinted models of human skin. Sci. Adv..

[28] Geng X., Qi Y., Liu X., Shi Y., Li H., Zhao L. (2022). A multifunctional antibacterial and self-healing hydrogel laden with bone marrow mesenchymal stem cell-derived exosomes for accelerating diabetic wound healing. Biomater. Adv..

[29] Han X., Saengow C., Ju L., Ren W., Ewoldt R.H., Irudayaraj J. (2024). Exosome-coated oxygen nanobubble-laden hydrogel augments intracellular delivery of exosomes for enhanced wound healing. Nat. Commun..

[30] Yang Y., Zhang J., Wu S., Deng Y., Wang S., Xie L. (2024). Exosome/antimicrobial peptide laden hydrogel wound dressings promote scarless wound healing through miR-21-5p-mediated multiple functions. Biomaterials.

[31] Li W., Wu S., Ren L., Feng B., Chen Z., Li Z. (2023). Development of an antiswelling hydrogel system incorporating M2-exosomes and photothermal effect for diabetic wound healing. ACS Nano.

[32] Dutta S.D., Hexiu J., Patel D.K., Ganguly K., Lim K.-T. (2021). 3D-printed bioactive and biodegradable hydrogel scaffolds of alginate/gelatin/cellulose nanocrystals for tissue engineering. Int. J. Biol. Macromol..

[33] Kim B.S., Kwon Y.W., Kong J.-S., Park G.T., Gao G., Han W. (2018). 3D cell printing of in vitro stabilized skin model and in vivo pre-vascularized skin patch using tissue-specific extracellular matrix bioink: a step towards advanced skin tissue engineering. Biomaterials.

[34] Pati F., Jang J., Ha D.-H., Won Kim S., Rhie J.-W., Shim J.-H. (2014). Printing three-dimensional tissue analogues with decellularized extracellular matrix bioink. Nat. Commun..

[35] Li M., Wang T., Tian H., Wei G., Zhao L., Shi Y. (2019). Macrophage-derived exosomes accelerate wound healing through their anti-inflammation effects in a diabetic rat model. Artif. Cell Nanomed. Biotechnol..

[36] Brown B.N., Ratner B.D., Goodman S.B., Amar S., Badylak S.F. (2012). Macrophage polarization: an opportunity for improved outcomes in biomaterials and regenerative medicine. Biomaterials.

[37] Cha B.H., Shin S.R., Leijten J., Li Y.C., Singh S., Liu J.C. (2017). Integrin‐mediated interactions control macrophage polarization in 3D hydrogels. Adv. Healthcare Mater..

[38] Mahon O.R., Browe D.C., Gonzalez-Fernandez T., Pitacco P., Whelan I.T., Von Euw S. (2020). Nano-particle mediated M2 macrophage polarization enhances bone formation and MSC osteogenesis in an IL-10 dependent manner. Biomaterials.

[39] Kwak G., Cheng J., Kim H., Song S., Lee S.J., Yang Y. (2022). Sustained exosome‐guided macrophage polarization using hydrolytically degradable PEG hydrogels for cutaneous wound healing: identification of key proteins and MiRNAs, and sustained release formulation. Small.

[40] Tu Z., Chen M., Wang M., Shao Z., Jiang X., Wang K. (2021). Engineering bioactive M2 macrophage‐polarized anti‐inflammatory, antioxidant, and antibacterial scaffolds for rapid angiogenesis and diabetic wound repair. Adv. Funct. Mater..

[41] Xie X., Nie H., Zhou Y., Lian S., Mei H., Lu Y. (2019). Eliminating blood oncogenic exosomes into the small intestine with aptamer-functionalized nanoparticles. Nat. Commun..

[42] Liu S., Chen X., Bao L., Liu T., Yuan P., Yang X. (2020). Treatment of infarcted heart tissue via the capture and local delivery of circulating exosomes through antibody-conjugated magnetic nanoparticles. Nat. Biomed. Eng..

[43] Tao S.-C., Yuan T., Zhang Y.-L., Yin W.-J., Guo S.-C., Zhang C.-Q. (2017). Exosomes derived from miR-140-5p-overexpressing human synovial mesenchymal stem cells enhance cartilage tissue regeneration and prevent osteoarthritis of the knee in a rat model. Theranostics.

[44] Sun Y., Zhang B., Zhai D., Wu C. (2021). Three-dimensional printing of bioceramic-induced macrophage exosomes: immunomodulation and osteogenesis/angiogenesis. NPG Asia Mater..

[45] Yang Y., Guo Z., Chen W., Wang X., Cao M., Han X. (2021). M2 macrophage-derived exosomes promote angiogenesis and growth of pancreatic ductal adenocarcinoma by targeting E2F2. Mol. Ther..

[46] Wang K., Dong R., Tang J., Li H., Dang J., Zhang Z. (2022). Exosomes laden self-healing injectable hydrogel enhances diabetic wound healing via regulating macrophage polarization to accelerate angiogenesis. Chem. Eng. J..

[47] Liu P., Xiong Y., Chen L., Lin C., Yang Y., Lin Z. (2022). Angiogenesis-based diabetic skin reconstruction through multifunctional hydrogel with sustained releasing of M2 Macrophage-derived exosome. Chem. Eng. J..

[48] Kim B.S., Kim H., Gao G., Jang J., Cho D.-W. (2017). Decellularized extracellular matrix: a step towards the next generation source for bioink manufacturing. Biofabrication.

[49] Williams C., Liao J., Joyce E., Wang B., Leach J., Sacks M. (2009). Altered structural and mechanical properties in decellularized rabbit carotid arteries. Acta Biomater..

[50] Lee K.I., Lee J.S., Kim J.G., Kang K.T., Jang J.W., Shim Y.B. (2013). Mechanical properties of decellularized tendon cultured by cyclic straining bioreactor. J. Biomed. Mater. Res., Part A.

[51] Den Hondt M., Vanaudenaerde B., Maughan E., Butler C., Crowley C., Verbeken E. (2017). An optimized non-destructive protocol for testing mechanical properties in decellularized rabbit trachea. Acta Biomater..

[52] Hinton T.J., Jallerat Q., Palchesko R.N., Park J.H., Grodzicki M.S., Shue H.-J. (2015). Three-dimensional printing of complex biological structures by freeform reversible embedding of suspended hydrogels. Sci. Adv..

[53] Hua W., Mitchell K., Raymond L., Godina B., Zhao D., Zhou W. (2021). Fluid bath-assisted 3D printing for biomedical applications: from pre-to postprinting stages. ACS Biomater. Sci. Eng..

[54] Colly A., Marquette C., Courtial E.-J. (2021). Poloxamer/poly (ethylene glycol) self-healing hydrogel for high-precision freeform reversible embedding of suspended hydrogel. Langmuir.

[55] Xiong Y., Chen L., Liu P., Yu T., Lin C., Yan C. (2022). All‐in‐one: multifunctional hydrogel accelerates oxidative diabetic wound healing through timed‐release of exosome and fibroblast growth factor. Small.

[56] Dutta S.D., Bin J., Ganguly K., Patel D.K., Lim K.-T. (2021). Electromagnetic field-assisted cell-laden 3D printed poloxamer-407 hydrogel for enhanced osteogenesis. RSC Adv..

[57] Dutta S.D., Park T., Ganguly K., Patel D.K., Bin J., Kim M.-C. (2021). Evaluation of the sensing potential of stem cell-secreted proteins via a microchip device under electromagnetic field stimulation. ACS Appl. Bio Mater..

[58] Chen Z., Zhao D., Liu B., Nian G., Li X., Yin J. (2019). 3D printing of multifunctional hydrogels. Adv. Funct. Mater..

[59] Zhao J., Hussain M., Wang M., Li Z., He N. (2020). Embedded 3D printing of multi-internal surfaces of hydrogels. Addit. Manuf..

[60] Giobbe G.G., Crowley C., Luni C., Campinoti S., Khedr M., Kretzschmar K. (2019). Extracellular matrix hydrogel derived from decellularized tissues enables endodermal organoid culture. Nat. Commun..

[61] Dutta S.D., Ganguly K., Randhawa A., Patil T., Patel D.K., Lim K.-T. (2023). Electrically stimulated 3D bioprinting of gelatin-polypyrrole hydrogel with dynamic semi-IPN network induces osteogenesis via collective signaling and immunopolarization. Biomaterials.

[62] Jara C.P., Wang O., do Prado T.P., Ismail A., Fabian F.M., Li H. (2020). Novel fibrin-fibronectin matrix accelerates mice skin wound healing. Bioact. Mater..

[63] Talbott H.E., Mascharak S., Griffin M., Wan D.C., Longaker M.T. (2022). Wound healing, fibroblast heterogeneity, and fibrosis. Cell Stem Cell.

[64] Koike Y., Yozaki M., Utani A., Murota H. (2020). Fibroblast growth factor 2 accelerates the epithelial–mesenchymal transition in keratinocytes during wound healing process. Sci. Rep..

[65] Zhou X., Brown B.A., Siegel A.P., El Masry M.S., Zeng X., Song W. (2020). Exosome-mediated crosstalk between keratinocytes and macrophages in cutaneous wound healing. ACS Nano.

[66] Toulza E., Mattiuzzo N.R., Galliano M.-F., Jonca N., Dossat C., Jacob D. (2007). Large-scale identification of human genes implicated in epidermal barrier function. Genome Biol..

[67] Sherrill J.D., Finlay D., Binder R.L., Robinson M.K., Wei X., Tiesman J.P. (2021). Transcriptomic analysis of human skin wound healing and rejuvenation following ablative fractional laser treatment. PLoS One.

[68] An G., Guo F., Liu X., Wang Z., Zhu Y., Fan Y. (2020). Functional reconstruction of injured corpus cavernosa using 3D-printed hydrogel scaffolds seeded with HIF-1α-expressing stem cells. Nat. Commun..

[69] Guo L., Niu X., Chen X., Lu F., Gao J., Chang Q. (2022). 3D direct writing egg white hydrogel promotes diabetic chronic wound healing via self-relied bioactive property. Biomaterials.

[70] Peng H., Li H., Zhang X., Tang J., Liang Y., Qiao L. (2023). 3D-exosomes laden multifunctional hydrogel enhances diabetic wound healing via accelerated angiogenesis. Chem. Eng. J..

[71] Shiekh P.A., Singh A., Kumar A. (2020). Exosome laden oxygen releasing antioxidant and antibacterial cryogel wound dressing OxOBand alleviate diabetic and infectious wound healing. Biomaterials.

[72] Jiang S., Zhuang Y., Cai M., Wang X., Lin K. (2023). Decellularized extracellular matrix: a promising strategy for skin repair and regeneration. Eng. Regen..

[73] Feng Y., Sun Z.-L., Liu S.-Y., Wu J.-J., Zhao B.-H., Lv G.-Z. (2019). Direct and indirect roles of macrophages in hypertrophic scar formation. Front. Physiol..

[74] Eming S.A., Martin P., Tomic-Canic M. (2014). Wound repair and regeneration: mechanisms, signaling, and translation. Sci. Transl. Med..

[75] Ji S., Li Y., Xiang L., Liu M., Xiong M., Cui W. (2024). Cocktail cell‐reprogrammed hydrogel microspheres achieving scarless hair follicle regeneration. Adv. Sci..

[76] Mok K.-W., Saxena N., Heitman N., Grisanti L., Srivastava D., Muraro M.J. (2019). Dermal condensate niche fate specification occurs prior to formation and is placode progenitor dependent. Dev. Cell.

[77] Kalekar L.A., Cohen J.N., Prevel N., Sandoval P.M., Mathur A.N., Moreau J.M. (2019). Regulatory T cells in skin are uniquely poised to suppress profibrotic immune responses. Sci. Immunol..

[78] Shook B.A., Wasko R.R., Rivera-Gonzalez G.C., Salazar-Gatzimas E., López-Giráldez F., Dash B.C. (2018). Myofibroblast proliferation and heterogeneity are supported by macrophages during skin repair. Science.

[79] Chen L., Li Z., Zheng Y., Zhou F., Zhao J., Zhai Q. (2022). 3D-printed dermis-specific extracellular matrix mitigates scar contraction via inducing early angiogenesis and macrophage M2 polarization. Bioact. Mater..

[80] Hu Y., Xiong Y., Zhu Y., Zhou F., Liu X., Chen S. (2023). Copper-Epigallocatechin gallate enhances therapeutic effects of 3D-printed dermal scaffolds in mitigating diabetic wound scarring. ACS Appl. Mater. Interfaces.

