# Supplementary material for: 3D bioprinting of engineered exosomes secreted from M2-polarized macrophages through immunomodulatory biomaterial promotes in vivo wound healing and angiogenesis
Source: Bioact Mater. 2024 Nov 27;45:345–62. doi: 10.1016/j.bioactmat.2024.11.026 (PMC11636135; doi:10.1016/j.bioactmat.2024.11.026)
Supplement: Multimedia component 1 [file mmc1.docx]

**Supporting Information for:**

**3D bioprinting of engineered exosomes secreted from M2-polarized macrophages through immunomodulatory biomaterial promotes *in vivo* wound healing and angiogenesis**

Sayan Deb Dutta,^1,2,3†^ Jeong Man An,^4†^ Jin Hexiu,^5†^ Aayushi Randhawa,^1,6†^ Keya Ganguly,^1^ Tejal V. Patil,^1,6^ Thavasyappan Thambi,^7^ Jangho Kim,^8*^ Yong-kyu Lee,^9*^ and Ki-Taek Lim^1,2,6*^

*^1^Department of Biosystems Engineering, Kangwon National University, Chuncheon-24341, Republic of Korea.*

*^2^Institute of Forest Science, Kangwon National University, Chuncheon-24341, Republic of Korea.*

*^3^School of Medicine, University of California Davis, Sacramento-95817, United States.*

*^4^Department of Bioengineering, College of Engineering, Hanyang University, Seoul-04763, Republic of Korea.*

*^5^Department of Plastic and Traumatic Surgery, Capital Medical University, Beijing-100069, China.*

*^6^Interdisciplinary Program in Smart Agriculture, Kangwon National University, Chuncheon-24341, Republic of Korea.*

*^7^Graduate School of Biotechnology, College of Life Sciences, Kyung Hee University, Yongin-17104, Republic of Korea.*

*^8^Department of Convergence Biosystems Engineering, Chonnam National University, Gwangju-61186, Republic of Korea.*

*^9^Department of Chemical and Biological Engineering, Korea National University of Transportation, Chungju-27470, Republic of Korea.*

^†^These four authors have contributed equally to this manuscript

**^*^Author for Correspondence:**

[rain2000@jnu.ac.kr](mailto:rain2000@jnu.ac.kr), [leeyk@ut.ac.kr](mailto:leeyk@ut.ac.kr), and [ktlim@kangwon.ac.kr](mailto:ktlim@kangwon.ac.kr)

**Contents:**

| **Supporting Methods** | 3-7 |
| --- | --- |
| **Supporting Results** | 7-11 |
| **Figure S1.** Characterization of PDA NSPs and scaffold fabrication | 12 |
| **Figure S2.** *In vitro* bioactivity evaluation of the scaffolds | 13 |
| **Figure S3.** Rheology and 3D printing of the fabricated Group-I (AGP) bioinks | 14 |
| **Figure S4.** Swelling efficiency of the 3D printed hydrogel scaffolds | 15 |
| **Figure S5.** Bioinformatics study and mechanisms of macrophage polarization | 16 |
| **Figure S6.** Cellular uptake study of exosomes | 17 |
| **Figure S7.** WST-8 assay of hKCs and hECs | 18 |
| **Figure S8.** Transwell cell migration study w/ or w/o exosomes | 19 |
| **Figure S9.** Characterization of the d-ECM hydrogel | 20 |
| **Figure S10.** Exosome labeling and time-resolved FL spectroscopy analysis | 21 |
| **Figure S11.** Angiogenic potential of the COL@d-ECM/Exo hydrogels | 22 |
| **Figure S12.** Transcriptomic study of the bioprinted skin model | 23 |
| **Figure S13.** Hemolysis assay | 24 |
| **Figure S14.** qRT-PCR analysis of *in vivo* wound healing after 14 days | 25 |
| **Figure S15.** Quality control data of scRNA-Seq study | 26 |
| **Table 1.** Comparative study of exosome-based bioprinted skin wound healing | 27 |
| **Table 1.** List of antibodies used in this study | 28 |
| **Table 2.** Gene primer list for RAW 264.7 cells | 29 |
| **Table 3.** Gene primer list for human (hDFs, hKCs, and hECs) cells | 30 |

**Supporting Methods**

**1. Exosome isolation and *in vitro* bioactivity evaluation**

**1.1. *Exosome isolation***

The exosomes from the control and treatment groups were isolated using the total exosome isolation kit (Thermo-Fischer Scientific, USA) according to the manufactures instruction. Briefly, the M2 polarized RAW 264.7 cells (5 × 10^4^/mL/well) were cultured in 3D printed AG and AGP-3 hydrogel scaffolds in 35 mm dish for 24 h in a serum free DMEM media. After that, the culture soup was collected and centrifuged at 800 × *g* to remove the cell debris. Next, the supernatant was mixed with the exosome isolation kit and the isolation was conducted according to the protocol. The cultivated exosomes from AG and AGP-3-treated groups were named as mExo-AG and mExo-AGP, respectively.

**1.2. *Characterizations***

The exosomes/microvesicles were characterized using nano tracking analysis (NTA), high-resolution TEM (HR-TEM), Western blotting (WB), and Dil staining. For WB analysis, control and M2 exosome proteins were lysed on ice with RIPA buffer, and the protein concentration determined using a BCA assay kit. Approximately 30 µg of whole protein lysate was subjected to sodium dodecyl sulfate-polyacrylamide gel electrophoresis (SDS-PAGE). The gel was then transferred to a polyvinylidene fluoride (PVDF) membrane (Millipore, USA) using standard procedures. Next, the membranes were blocked with 5% skim milk in Tris-buffer supplemented with 0.1% Tween-20 (TBST) for 1 h. After blocking, the membranes were incubated with specific antibodies against CD9 and CD63 at 4 °C for 24 h. The membranes were then incubated with horseradish peroxidase (HRP)-conjugated secondary antibodies, followed by application of luminol detection buffer (Santa Cruz Biotechnology, USA). Finally, the blots were visualized using an ECL detection system (Bio-Rad, USA). All primary and secondary antibodies were purchased from Abcam (Cambridge, USA). For Dil staining, freshly isolated exosomes were incubated with 5 µM Dil dye at 4 °C for 1 h. The solution was then centrifuged at 13,000 rpm for 10 min to remove unbound dye. The Dil-labelled exosomes were stored at 4 °C until further use.

**1.3. *Cellular uptake study***

An *in vitro* cellular uptake study of exosomes was conducted using hDFs after 2 h of incubation. Briefly, 0.5 × 10^4^ cells were incubated with FITC-labelled exosomes. After the desired time interval, the cells were washed with PBS and incubated with 10 µL of LysoTracker red (Thermo Fischer Scientific, USA) dye, and exosome uptake was visualized using a confocal laser microscope (CLSM, Zeiss, Germany) with appropriate lasers.

**1.4. *In vitro cell viability assays***

The *in vitro* cytotoxicity of exosomes on human cells (hDFs, hKCs, and hECs) was examined using the WST-8 assay. Briefly, 1.5 × 10^4^ cells were cultured in 96-well plates supplemented with DMEM media. hECs were cultured in EGM, as described in the cell culture section. After reaching 60% confluency, the cells were treated with 10 µL of mExo-Ctrl and mExo-AGP. Plates devoid of exosomes were used as negative controls. After 24 h, the cells were incubated with 10 µL of WST-8 dye for 2 h. After 2 h, the formazan produced was quantified using a spectrophotometer (TECAN Pro, Switzerland) at 450 nm. Data are reported as the mean ± s.d. of triplicate experiments (*n* = 3).

**1.5. *In vitro cell migration assay***

To examine the effects of M2 exosomes on hDFs migration, we performed a Transwell® cell migration assay. For this, 1.5 × 10^4^ cells were cultured in serum-free DMEM supplemented with 10 µL of mExo-Ctrl and mExo-AGP in the upper chamber (0.8 µm insert). The lower chamber was supplemented with serum DMEM media. After 24 h, the cells were fixed with 100% methanol and stained with Harris Hematoxylin solution. After staining, cells were washed with PBS and directly visualized using an inverted optical microscope. The images were captured with a 0.5× objective lens, and the data were analyzed using the NIH ImageJ software (v1.8, Bethesda, USA).

**1.6. *In vitro tube formation assay***

The angiogenic potential of M2 exosomes was assessed using a tube formation assay. Briefly, hMSCs (1.5 × 10^4^ cells) were incubated with or without exosomes in Matrigel-coated 24-well plates and cultured for three days. After the desired time point, cells were stained with an angiogenic marker (CD31) and visualized using an inverted fluorescence microscope (DMi8, Leica, Germany). Endothelial tube formation was photographed and quantified using ImageJ.

**2. *In vivo* wound healing study**

**2.1. *Subcutaneous wound model***

3-4 weeks old imprinted control region (ICR; SPF Biotechnology, Beijing, China) male rats (*N* = 15) were randomly divided into three groups: (1) Control group (devoid of scaffold; *n* = 5), (2) COL@d-ECM group (scaffold w/o Exo; *n* =5), and (3) COL@d-ECM + Exo (scaffold w/ Exo; *n* = 5). We expect the better *in vivo* wound healing and angiogenic potential of the COL@d-ECM + Exo group owing to its ability to accelerate the skin-related gene markers (*e.g*., *FN*, *COL*, and *KRTs*) and angiogenic marker (*e.g*., *VEGF* and *CD31*) expression. For the wound healing study, the dorsal skin of the rats was gently trimmed, followed by disinfection with 70% (*v*/*v*) ethanol. Next, the skin was slightly pushed towards the top side, and a subcutaneous wound of 10 mm diameter was carefully made using a sterile biopsy punch. After that, UV-sterilized 3D printed scaffolds with a 10 × 2 mm dimension were carefully implanted onto the wound area, which was covered with a sterile gauge. All the rats were kept in a soundproof room with gentle access to food and water. The wound healing rate (macroscopic healing) was photographed after 7 and 14 days of implantation.

**2.2. *Histological evaluation***

The microscopic wound healing was observed after 14 days of scaffold implantation. For this, the excised wound beds were fixed with 4% paraformaldehyde (PFA; Sigma-Aldrich, USA), followed by paraffin embedding and sectioning. Afterward, the slide-mounted tissue samples were stained with hematoxylin and eosin (H&E; Sigma-Aldrich, USA) to evaluate the epidermal thickness, re-epithelialization, and the presence or absence of inflammatory cells. In this study, the stained slides were evaluated as following the inflammation score scale: 1 = no inflammation, 2 = minimal inflammation, 3 = moderate inflammation, and 4 = higher inflammation. Massion’s Trichrome staining was performed after 14 days of scaffold implantation to evaluate the collagen formation in the wound bed. All the experiments were approved by the Capital Medical University Animal Experimental Ethics Committee (Permission No: KQYY-202012-004), Beijing, China. The experiments were performed in a blinded fashion.

**2.3. *Isolation of cells from the wound bed and qRT-PCR analysis***

The single-cell suspension was prepared to evaluate the level of inflammatory gene markers (*IL-6* and *TNF-α*) during the wound healing process. Briefly, 14-day wound bed skin samples were cut into 2 mm^2^ small pieces using a biopsy punch and subsequently washed with 1× PBS. After washing, the tissue samples were digested with 0.3% dispase-II enzyme (Sigma-Aldrich, USA) at 37 °C for 90 min. The dermis samples were digested with a balanced buffer containing collagenase D (1 mg/mL, Sigma-Aldrich, USA), hyaluronidase (1 mg/mL, Sigma-Aldrich, USA), and DNase (150 U/mL, Sigma-Aldrich, USA) in a water bath with a constant temperature of 37 °C for 2-3 h. The digested samples were filtered with a 70 µm strainer (Corning, USA) and centrifuged to get cell pellets. The pellet was resuspended in 1× PBS and centrifuged to discard any debris. The as-obtained cell pellet was directly used for Trizol-based RNA extraction (Sigma-Aldrich, USA) and qRT-PCR analysis.

**2.4. *Library preparation and scRNA sequencing***

At various time points, the skin tissues from the wound bed were collected without separating the epidermis and dermis. The excised tissue samples were washed with 1× PBS and digested using a digestion media containing DMEM, containing 10% FBS, 200 mg/mL collagenase-IV (Gibco-BRL, USA), 1 mg/mL DNase (Takara Bio, Japan), and 25 mM HEPES buffer (Welgene, Republic of Korea) and incubated in a shaking water bath at 37 °C for 2 h. After that, the digested solution was strained through a 40 $\mu$m cell strainer (Corning^®^) to generate a single-cell suspension. The CD45^+^ single-cells from each organ of different groups were directly used for library preparation and single-cell RNA (scRNA-Seq) sequencing analysis. Briefly, the cells were counted (~1.2 × 10^4^ ml^-1^), suspended in 0.04% BSA (prepared in 1× PBS), and processed with a Chromium single-cell 3*'* library kit and gel bead kit (10X Genomics) according to the manufacturer’s guidelines. After desirable lysis and treatment, the single-cell gene libraries were sequenced using the NovaSeq6000 (Illumina) sequencer at a reading depth of ~15,000 reads per cell from total expression libraries and 12,000 reads per cell using TCR libraries.

The raw sequencing data from Illumina was processed using the *Cell Ranger* (v3.0.1, 10X Genomics) with standard algorithms, followed by statistical analysis. Next, the FASTQ files were then processed with the *Seurat* (v5.1) R package for clustering and visualization and compared using a mouse mm39 (*Mus musculus*, GCF_000001635.27) reference genome with a threshold of >5000 variables. The obtained data was plotted using the *Uniform Manifold Approximation and Projection* (UMAP) clustering with *k*-nearest neighboring (KNN) analysis to identify the differentially expressed genes associated with macrophage polarization in the wound tissue after implantation of various samples *in-vivo*. The defined clusters were then screened for the macrophage polarization markers, and their gene expression was studied. The expression of major macrophage polarization (M1 and M2) genes was plotted using the *Heatmapper* R package (RStudio). The Gene Set Enrichment Analysis (GSEA v4.0.2) was analyzed to understand the significant up-regulation/down-regulation pathways in various groups.

**2.5. *Immunocytochemistry***

The *in vivo* macrophage polarization potential of the fabricated COL@d-ECM/M2-Exo was evaluated using immunocytochemistry after 3, 5, 7, and 14 days of wound healing. We used canonical M1 (CD86 and NOS2) and M2 (CD163 and CD206) markers to evaluate the macrophage polarization and validation of the scRNA-Seq data. Following implantation, at various time points, the wound tissue was collected and stained with respective primary and secondary antibodies. The stained slides were visualized using an inverted fluorescence microscope (Axiovert 5, Zeiss, Germany) with a 40× objective lens. The images were accrued using ZEN software and processed with ImageJ (v1.8, NIH, Bethesda, USA) for intensity measurements. The detailed information of antibodies is given in **Table S2**.

**Supporting Results**

**1. Characterization of the nanocomposite bioinks**

PDA NSPs were synthesized *via* pH-induced polymerization by controlling the reaction time. In an alkaline medium, several dopamine molecules undergo covalent oxidative polymerization, in which the catechol groups of dopamine are oxidized to dopamine-benzoquinone and 5,6-dihydroxyindole (DHI). In the next step, several DHI molecules self-assemble to form dimeric or trimeric conjugates, which ultimately gave rise to PDA NSPs [[35](#_ENREF_35)]. The morphology of the PDA NSPs was investigated through scanning electron microscopy (FE-SEM) and high-resolution transmission electron microscopy (HR-TEM) **(Figure S1)**. As shown in **Figure S1(a)**, the PDA NSPs exhibited smooth and spherical to oval-shaped morphology with an average diameter of ~ 0.5 - 2.4 ± 0.6 µm. To examine the intrinsic structure and elemental composition, we performed energy-dispersive X-ray spectroscopy (EDS). In accordance with the SEM data, the average size of the PDA particles was found to be 1.5 - 2.2 ± 1.2 µm. EDX analysis revealed the presence of mostly carbon (C) and oxygen (O) compared to other metallic elements (P or Ca). The surface functional groups of the PDA NSPs were monitored using Fourier-transform infrared spectroscopy (FT-IR) **(Figure S1(b))**. Pure dopamine exhibited a characteristic stretching vibration at 3326 cm^-1^ owing to the presence of aromatic hydroxyl (-OH) groups. Several peaks at 1174, 1288, 1319, and 1497 cm^-1^ are attributed to the presence of C-C, -C-O, hydroxyl (-OH), and amide (N-H) stretching in the dopamine backbone [[36](#_ENREF_36), [37](#_ENREF_37)]. Notably, the peak around 3000 to 3300 cm^-1^ completely disappeared in the PDA NSPs and showed a broad peak owing to the oxidation and fusion of several DHI molecules. Moreover, two characteristic peaks at approximately 1148-1190 cm^-1^ and 1501-1590 cm^-1^ appeared in the PDA NSPs owing to the C-N bending vibration of aromatic indolequinone and C=C of aromatic indole, respectively [[37-39](#_ENREF_37)]. Thus, FT-IR spectroscopy confirmed that the indole and indolequinone moieties of PDA were absent in pure dopamine.

Next, we used PDA NSPs to fabricate a composite bioink for macrophage polarization. A hybrid bioink was prepared using alginate (A) and gelatin (G) as polymer sources and PDA NSPs (P) as nano-fillers to provide a hydrophilic and adhesive matrix for macrophage cell adhesion and proliferation. The bioink was characterized using FT-IR and X-ray diffraction (XRD) to study the chemical interactions and structural changes. As shown in **Figure S1(c)**, the pure AG bioink exhibited three characteristic peaks at around 1628, 1541, and 1229 cm^-1^, attributed to the presence of Amide-I (C=O and C-N stretching vibrations), Amide-II, and Amide-III (N-H and C-N stretching vibrations) regions blended with the symmetric and asymmetric stretching vibrations of carboxylic (-C=O) moieties, respectively. Interestingly, after PDA incorporation, the peak at approximately 1628 cm^-1^ was slightly shifted to a higher wavenumber (1630 to 1634 cm^-1^) as the concentration of PDA NSPs increased, indicating possible interaction between AG and PDA NSPs via hydrogen bond formation.

Moreover, the stretching vibration at 1083 cm^-1^ of AG was slightly shifted to a higher wavenumber (~1091 cm^-1^), which was attributed to the C-N stretching vibration of alginate/polydopamine. Thus, from the FT-IR results, we surmised that the PDA NSPs mainly interacted with the AG matrix via hydrogen bond formation with gelatin and C-N bonds with the alginate backbone. Next, we examined the structural changes in the hydrogel scaffold using XRD analysis. As shown in **Figure S1(d)**, the pure AG scaffold exhibits a broad peak at approximately 22.7°, followed by a sharp peak at 32°. Interestingly, as the concentration of PDA was increased, the peak at 22.7° slightly shifted to a lower angle (22.7°$\to$21.7°) with a relatively narrow peak, indicating that PDA incorporation slightly increased the crystallinity owing to the homogenous dispersion into the AG matrix.

The mineral adsorption efficiency of the fabricated scaffolds was investigated *in vitro* using simulated body fluid (SBF, pH 7.4) over 14 d of soaking. As shown in **Figure S2(a)**, the pure AG scaffold exhibited a porous morphology with a smooth surface. Notably, after PDA incorporation, the scaffolds (AGP-1, AGP-2, and AGP-3) exhibited rough and angular protrusions on their surfaces. After 14 days of SBF soaking, the AGP scaffolds exhibited a dramatic increase in mineral deposition compared with the pure AG scaffold, which was characterized by the presence of crystal deposits on the scaffold surface. The mineralization process usually involves the dissolution of calcium (Ca) and phosphate (P) via nucleation-mediated growth on the PDA substrate [[40](#_ENREF_40), [41](#_ENREF_41)]. We observed that as the concentration of PDA increased in the AG matrix, the Ca/P deposition was found to be significantly higher (^*^*p* < 0.05, Ca/P ratio = 5.18 wt. %) **(Figure S2(b))**. The schematic illustration of the Ca/P binding through the AGP scaffold surface is shown in **Figure S2(c)**.

**2. Rheological investigation of the AGP bioinks**

Rheology is an important tool for investigating the properties of bioinks property for 3D printing applications [[42-44](#_ENREF_42)]. The viscoelastic properties of the AGP bioinks (bioinks for macrophage phenotyping and Exo cultivation) were measured using a rotational rheometer with an 8 mm parallel plate at room temperature (25 °C). The storage modulus (G′), loss modulus (G′′), and complex viscosity (η*) were measured within the frequency range of 0.1 to 100 Rad s^-1^ with a 1% strain rate at 1 Hz. As shown in **Figure S3(a)**, G′ increased as the concentration of PDA NSPs increased. Notably, the AGP-3 hydrogel exhibited the highest G′ value compared to that of the pure AG hydrogel. This was attributed to the strong interaction and tougher inner-connecting polymeric network (IPN) after PDA insertion and higher dispersion stability, resulting in decreased motion of the polymer chain, followed by a decrease in G′′ values [[45](#_ENREF_45)]. The G′ values of pure AG, AGP-1, AGP-2, and AGP-3 at 100 Rad s^-1^ were measured as 5214 Pa, 32902 Pa, 94749 Pa, and 451198 Pa, respectively. Similarly, the G′′ values of pure AG, AGP-1, AGP-2, and AGP-3 at 100 Rad s^-1^ were measured as 2053, 7394, 8028, and 23593 Pa, respectively **(Figure S3(b))**. Next, we examined the viscosity of the bioinks at angular frequency ranging from 0.1 to 100 Rad s^-1^ at room temperature. It is interesting to note that all bioinks exhibited shear-induced viscosity changes within the measured range. We observed an initial increase in viscosity at 0.1 Rad s^-1^, followed by a sharp decrease as the frequency was changed to 100 Rad s^-1^, suggesting the shear-thinning nature of the bioinks **(Figure S3(c))**. The η* value of the pure AG, AGP-1, AGP-2, and AGP-3 at 0.1 Rad s^-1^ was calculated to be 337.23, 531.19, 976.43, and 5582.86 Pa. s, respectively. Our results demonstrate that Group-1 bioinks are highly viscoelastic, making them suitable for 3D bioprinting applications.

Polydopamine-based hydrogels have been shown to have excellent printability owing to their self-healing and self-crosslinking nature [[46-48](#_ENREF_46)]. For 3D bioprinting, we used alginate/gelatin owing to its exceptional printability in a low-temperature environment. Gelatin, a hydrolysis product of collagen, shows temperature-dependent gelation properties, making it ideal for 3D bioprinting [[49](#_ENREF_49), [50](#_ENREF_50)]. In this study, we used 3% alginate and 2% gelatin (both *w/v*) for 3D printing. Prior to 3D printing, all the hydrogel inks were incubated at 4 °C to permit physical crosslinking. Subsequently, a 20 × 1 mm construct was designed using SolidWorks software and sliced using an open-source slicing tool. Subsequently, the printing cartridges were carefully loaded onto the printing device, and a 22G metal-head nozzle (length: 0.5′; inner diameter: 400 µm) was used for 3D printing **(Figure S3(d))**. Next, 3D printing was performed in a cooled print bed, maintaining a temperature of approximately 4-10 °C. After printing, the constructs were immersed in 100 mM CaCl_2_ solution to initiate alginate cross-linking, as reported in our previous study [[32](#_ENREF_32)]. Digital photographs of the 3D printing process and an image of the printed hydrogel after CaCl_2_ crosslinking are shown in **Figure S3(e)**. Both the AG and AGP-3 hydrogels showed good 3D printed morphology with unique porosity **(Figure S3(f))**. Because the degree of crosslinking has a potential impact on the swelling efficiency, we also tested the swelling performance of the 3D-printed hydrogel scaffolds after CaCl_2_ crosslinking. As shown in **Figure S4**, CaCl_2_ crosslinking had a negligible effect on the swelling efficiency. Owing to the presence of PDA, the AGP scaffolds exhibited greater swelling potential than the pure AG scaffold. Taken together, our results demonstrated that the Group-I bioinks had exceptional bioprinting performance, high viscoelasticity, and desirable swelling properties for macrophage adhesion, proliferation, and differentiation.

**
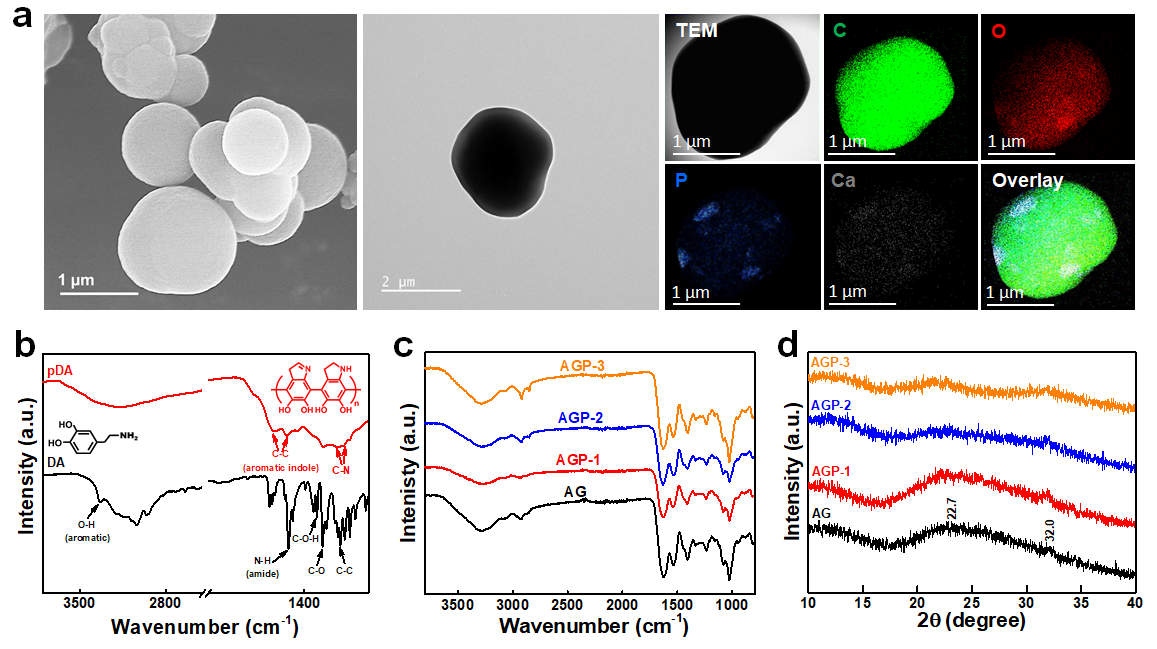
**

**Figure S1.** Characterization of the polydopamine nanospheres (PDA NSPs) and its composite hydrogel scaffolds. **(a)** HR-SEM image of the as-synthesized PDA NSPs. The HR-TEM with corresponding EDS mapping data of the PDA NSPs showing the presence of carbon (C), oxygen (O), phosphorus (P), and calcium (Ca). **(b)** FT-IR spectra of the pure dopamine (DA) and the PDA NSPs. **(c)** FT-IR spectra of the pure AG and its composite bioinks. **(d)** XRD pattern of the pure AG and its composite hydrogel scaffolds. Scale bar: 1 and 2 µm.

**
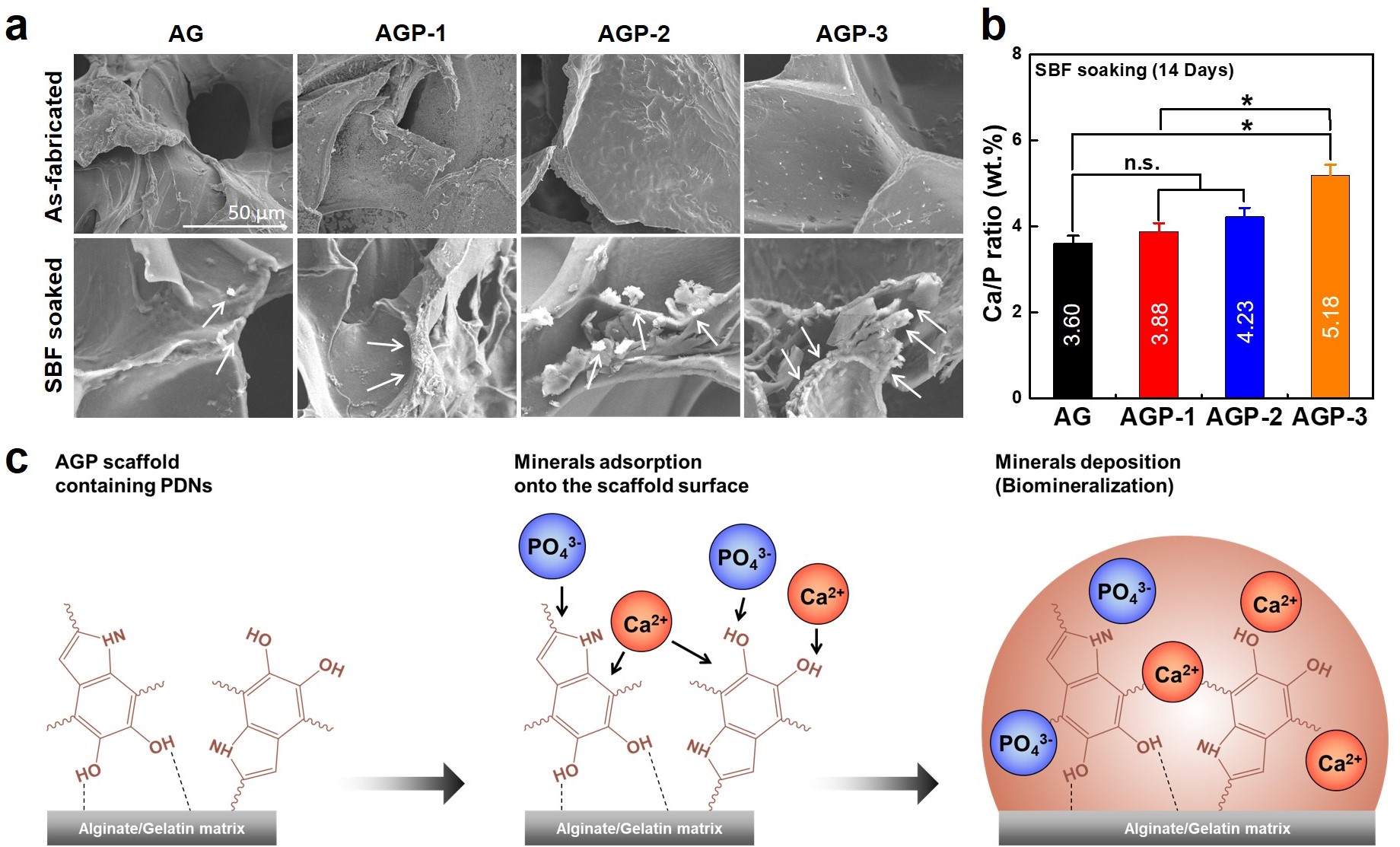
**

**Figure S2.** *In vitro* bioactivity evaluation of the polydopamine nanospheres (PDA NSPs/PDNs) and its composite hydrogel scaffolds. **(a)** *In vitro* mineral adsorption efficiency of the fabricated scaffolds in stimulated body fluid (SBF) after 14 days of incubation. **(b)** The Ca/P ratio after SBF soaking of the scaffolds. Scale bar: 50 µm. Data reported as mean ± s.d. of the triplicate experiments (*n* = 3), statistical significance at ^*^*p* < 0.05. **(c)** Schematic illustration for the AGP scaffold-induced biomineralization procedure.

**
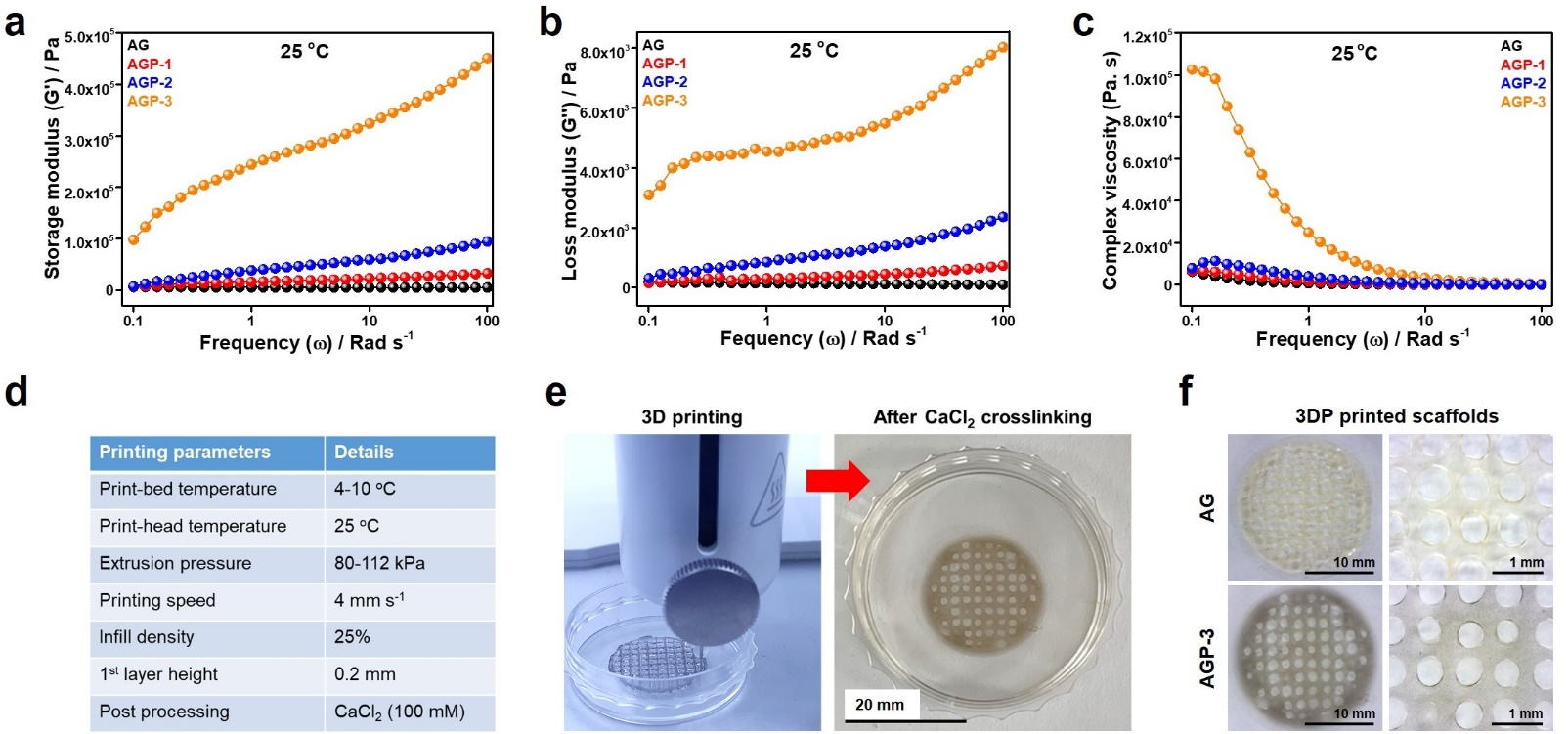
**

**Figure S3.** Viscoelastic property of the fabricated bioinks. **(a)** Representative storage modulus (G′) modulus of the pure AG and its composite bioinks. **(b)** The loss (G′′) modulus of the fabricated bioinks within frequency range of 0.1 to 100 Rad s^-1^. **(c)** The complex viscosity of the fabricated bioinks showing the shear thinning and thickening properties. **(d)** The 3D printing parameters used in this study. **(e)** Digital photographs of the 3D printing process using AGP-3 bioink and ionic crosslinking with 100 mM CaCl_2_ solution. **(f)** Digital photographs of the AG and AGP-3 hydrogel after 1^st^ layer of 3D printing. Scale bar: 1, 10, and 20 mm.

**
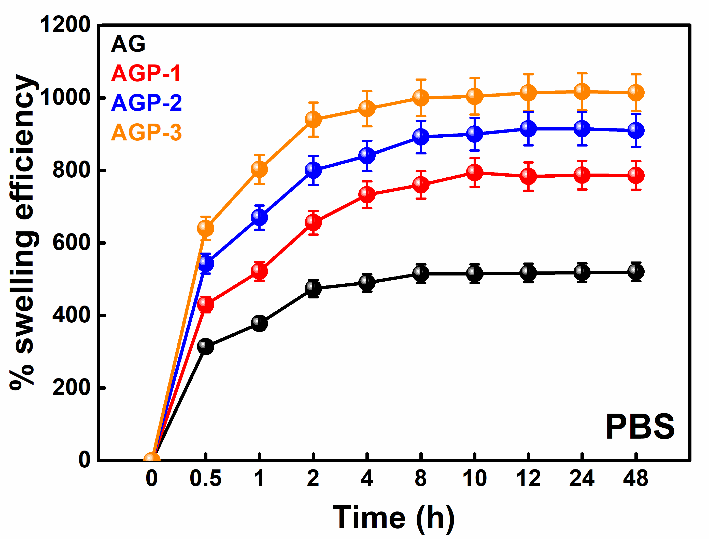
**

**Figure S4.** Swelling efficiency of the 3D printed hydrogel scaffolds at indicated time points.


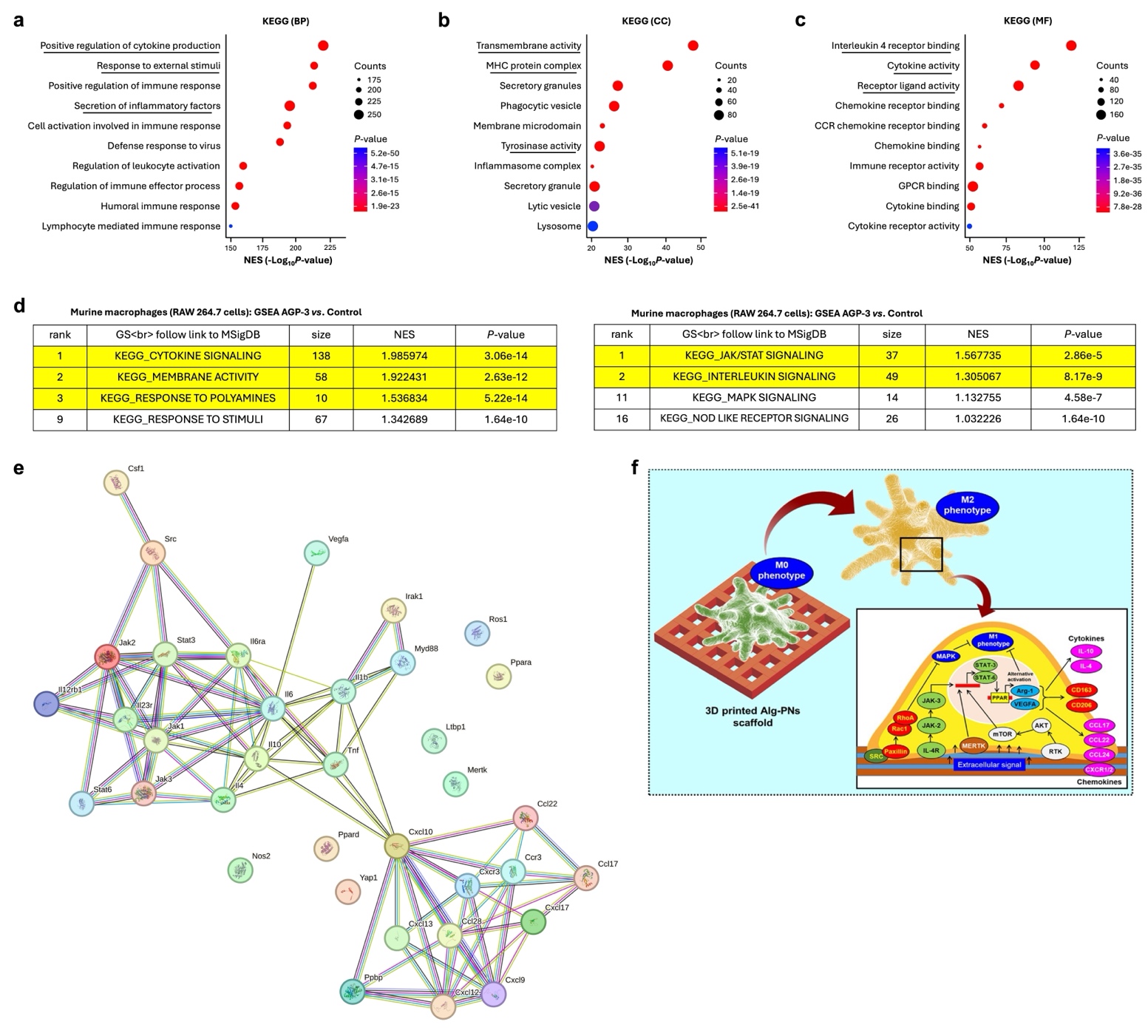


**Figure S5.** Bioinformatics study of the macrophage polarization using Alg/PNs scaffolds. **(a-c)** KEGG enrichment analysis (BP, CC, and MF) of DEGs associated with Cluster-C when compared between AGP-3 *vs*. Control, suggesting the activation of various immune-regulatory processes. The underlined processes with higher gene counts and enrichment were noted. **(d)** GSEA analysis showing the top-ranked genes associated with ‘cytokine signaling’ and ‘immune-regulatory pathways.’ **(e)** STRING protein-protein interaction map of the key identified genes involved in immune regulation of macrophages in Cluster-C. **(f)** Schematic illustration of the signaling pathways involved in scaffold-assisted M2 macrophage polarization of RAW264.7 cells.


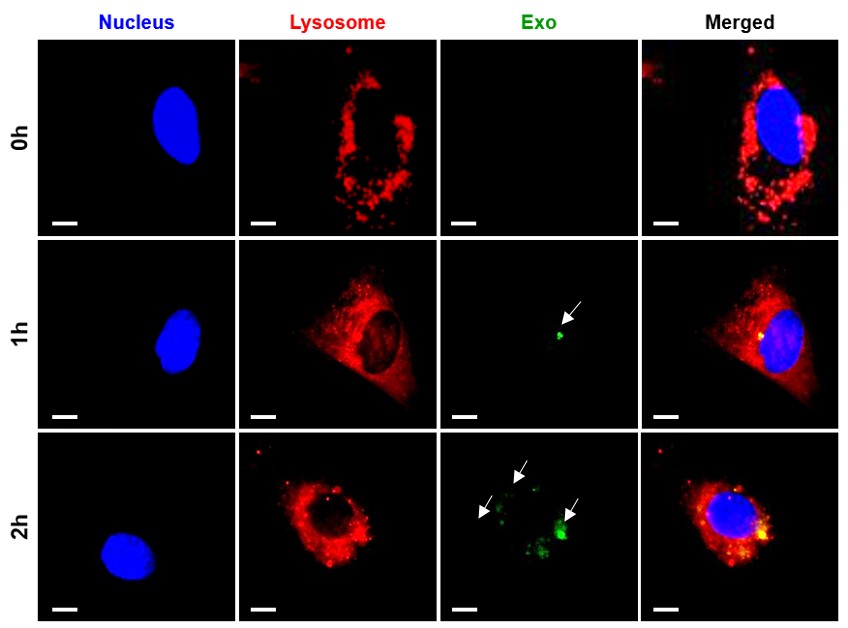


**Figure S6.** Representative confocal images of the hDFs showing the localization of mExo-AGP (green) with the lysosome (red) at indicated time points. Scale bar: 50 µm.


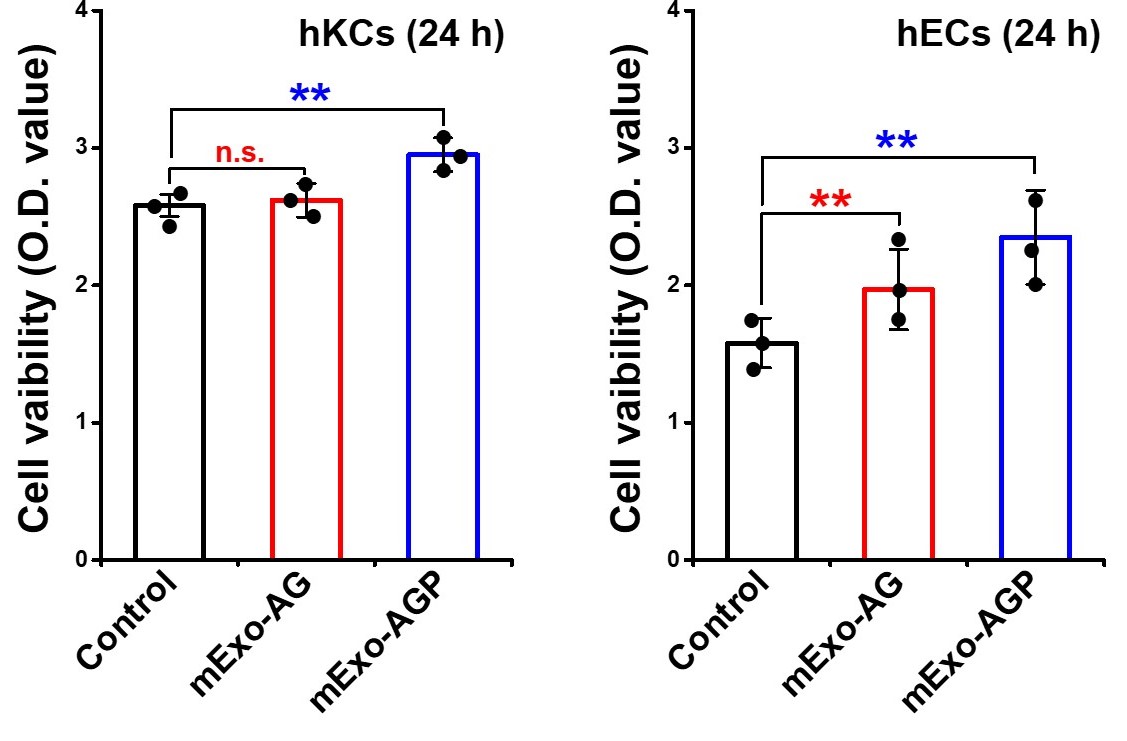


**Figure S7.** WST-8 assay showing the *in vitro* cytotoxicity profile of the exosomes in hKCs and hECs. Data reported are mean ± s.d. of triplicate experiments (*n* = 3), with statistical significance at ^**^*p* < 0.01 (One-way ANOVA test).

**
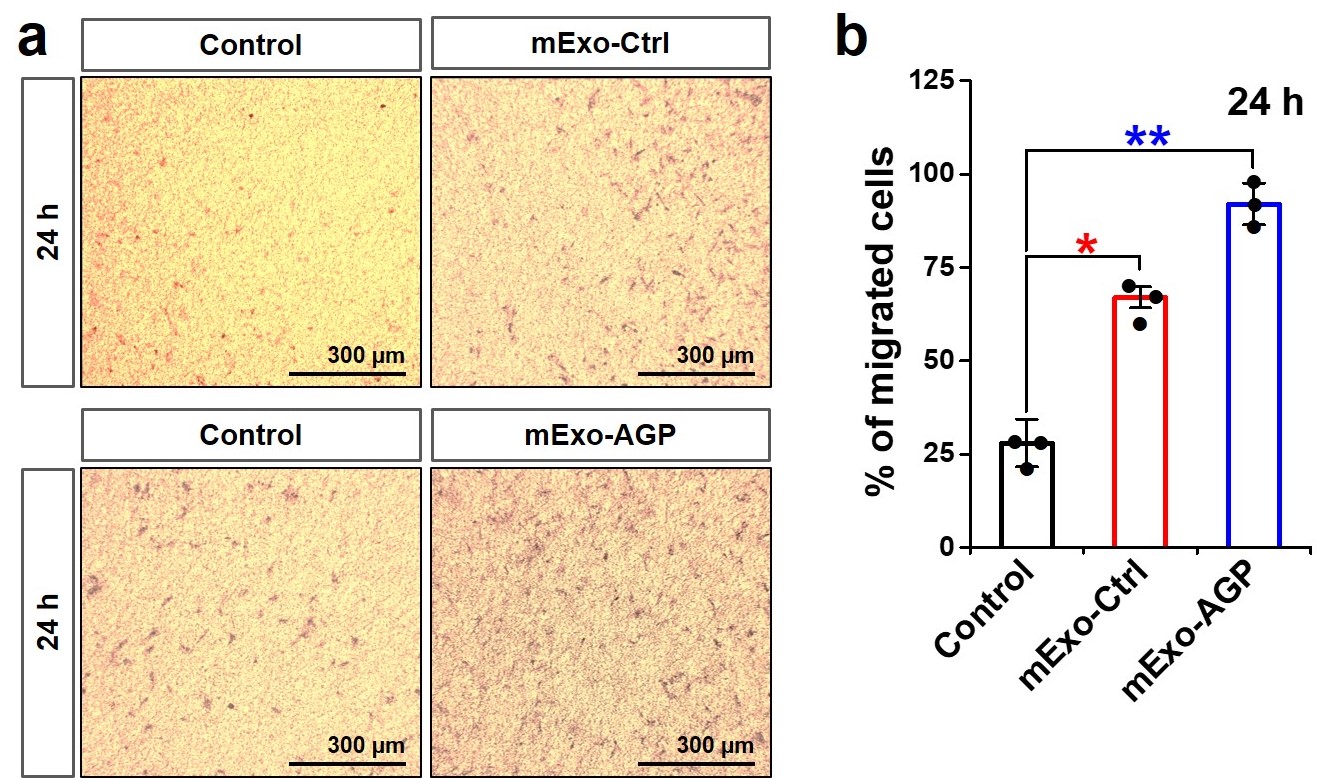
**

**Figure S8.** Transwell migration assay with corresponding quantification showing the effect of immunopolarized exosomes on cell migration. hDFs (passage-4) were used for cell migration study. Data reported are mean ± s.d. of triplicate experiments (*n* = 3), with statistical significance at ^*^*p* < 0.05 and ^**^*p* < 0.01 (One-way ANOVA test). Scale bar: 300 µm.


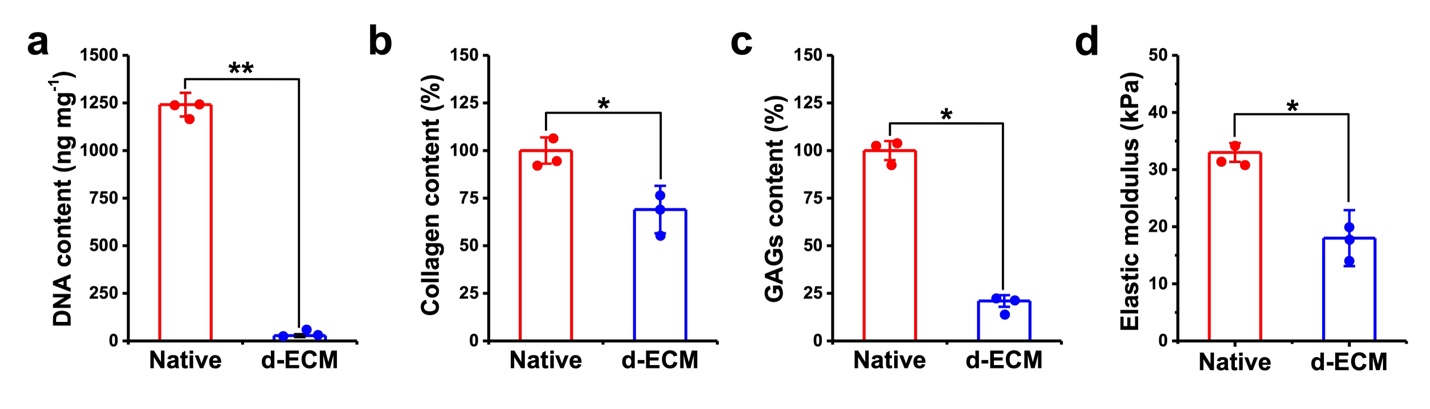


**Figure S9.** **(a-c)** Relative abundance of the nuclear (DNA) and ECM components (collagen and GAGs) in native tissue and d-ECM mat. **(f)** The elastic modulus of the native chicken skin and d-ECM mat. Data reported are mean ± s.d. of triplicate experiments (*n* = 3), with statistical significance at ^*^*p* < 0.05 and ^**^*p* < 0.01 (One-way ANOVA test).


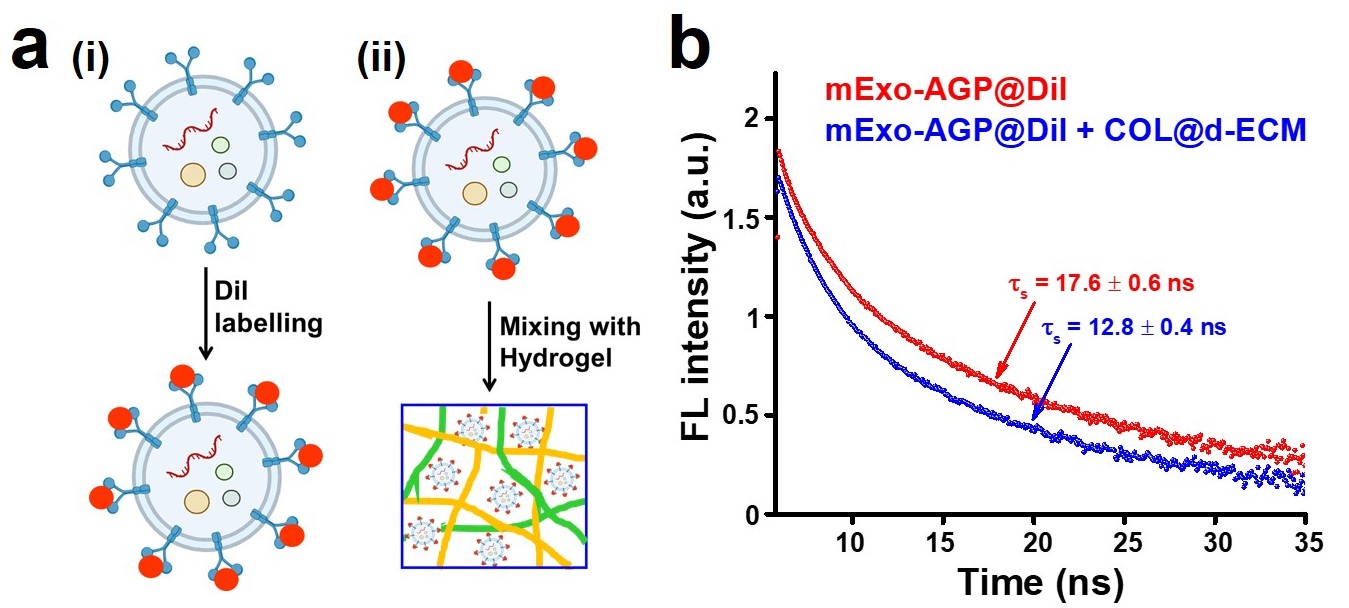


**Figure S10.** **(a)** Schematic illustration of the exosome labeling with Dil **(i)** and fabrication of the exosome-laden bioink **(ii).** **(b)** Time-resolved fluorescence spectroscopy analysis of the as-labelled exosome and exosome bounded to hydrogel ink.


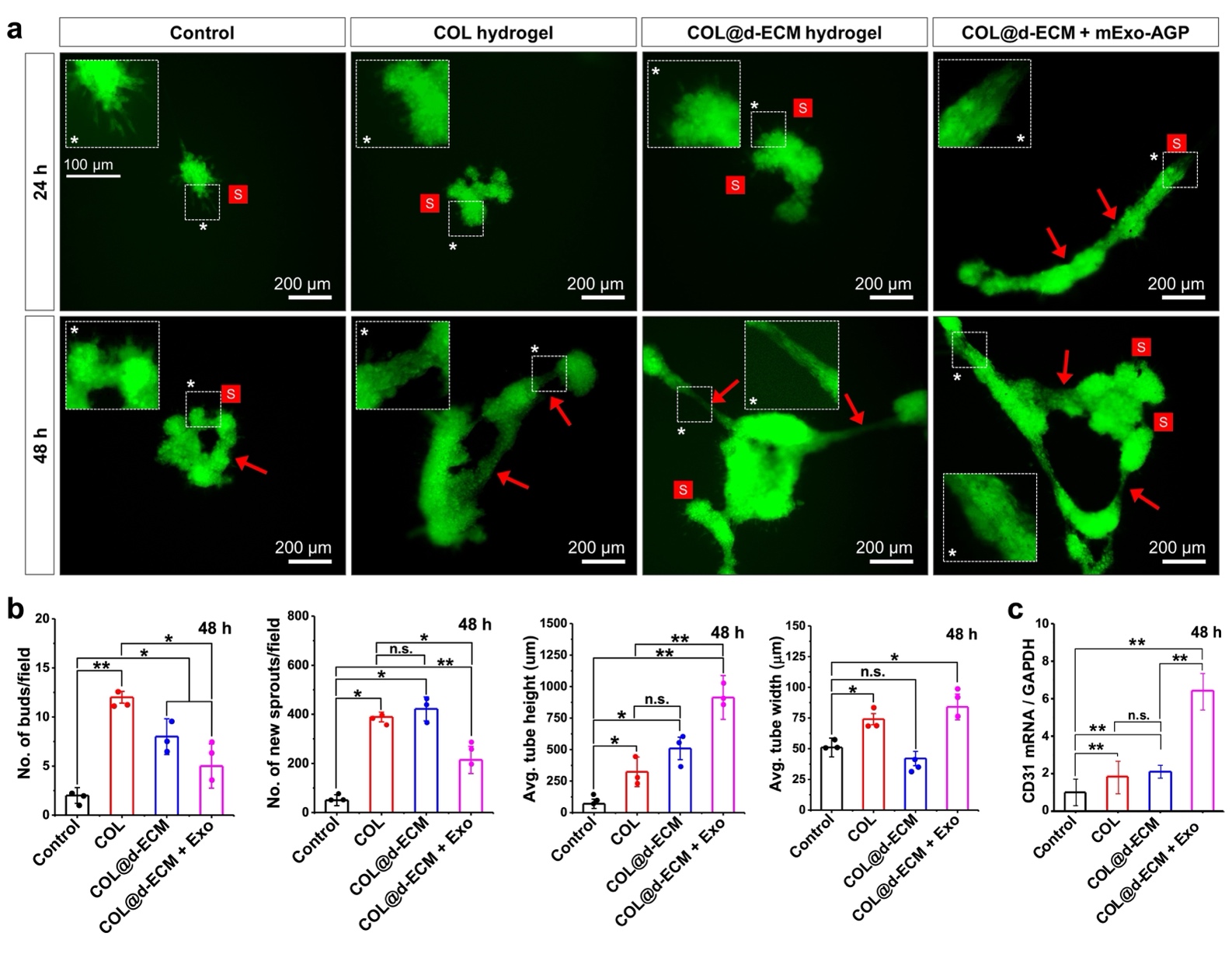


**Figure S11.** **(a)** FL microscopy images of the cells showing the endothelial tube morphology w/ or w/o exosome treatment. The cells were pre-stained with Cell Tracker Green dye to visualize the morphological changes. Scale bar: 100 and 200 µm. **(b)** The quantification data of the tube formation assay. **(c)** qRT-PCR analysis of CD31 gene markers under various treatment conditions of hBMSCs during tube formation assay. Data reported as mean ± s.d. of triplicate experiments, statistical significance at ^*^*p* < 0.05 and ^**^*p* < 0.01 (One-way ANOVA test). A value beyond the statistical parameter was considered as not significant (n.s.).

**
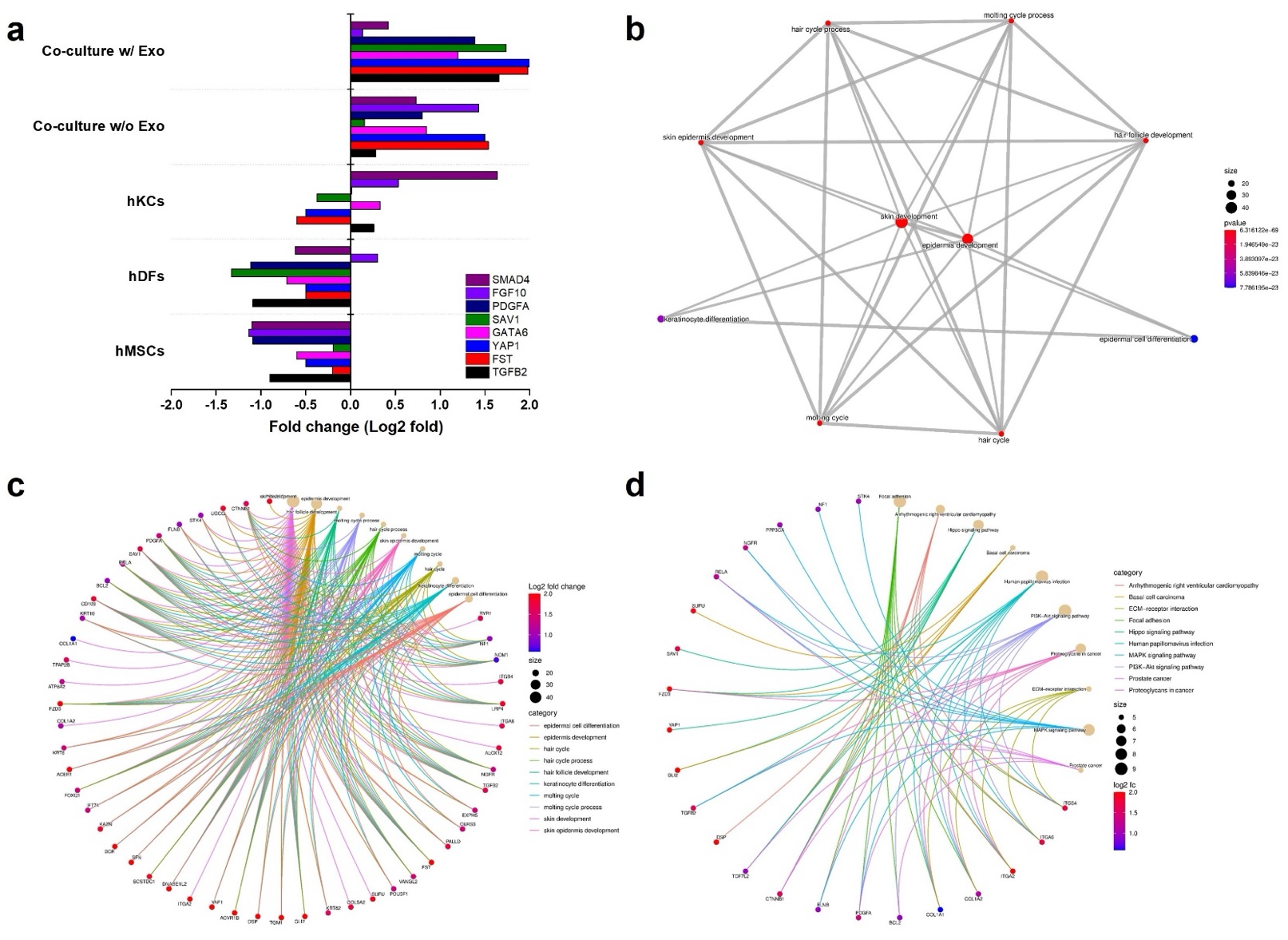
**

**Figure S12.** Transcriptomic changes of the bioprinted skin model. **(a)** The expression of DEGs in various signaling pathways is associated with ECM signaling, Hippo signaling, PI3K-Akt signaling, PDGF signaling, and TGF-β signaling. **(b)** GSEA analysis shows the enrichment of epidermal signaling in the presence of bioprinted COL@d-ECM + Exo hydrogel. **(c, d)** Cnet plot showing the functional enrichment (^*^*p* < 0.05) of various DEGs associated with epidermis development and its signaling pathways during 7 days of *in vitro* culture.

**

**

**Figure S13.** Blood biocompatibility of the pure and Exo-laden bioinks. *In vitro* hemolysis assay demonstrating the superior biosafety of the formulated bioinks. 0.5% Triton-X 100 and PBS were taken as negative and positive controls. Data reported as mean ± s.d. of the triplicate experiments (*n* = 3), statistical significance at ^*^*p* < 0.05.


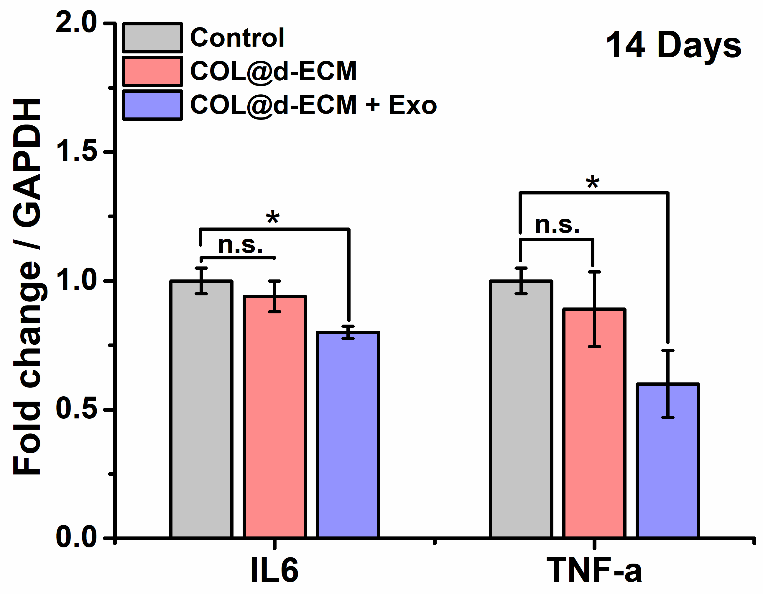


**Figure S14.** qRT-PCR analysis showing the expression of IL-6 and TNF-α gene markers of wound bed-derived cells after 14 days post-implantation. Data are mean ± s.d. of triplicate experiments (*n* = 3), with statistical significance at ^*^*p* < 0.05 (One-way ANOVA test).


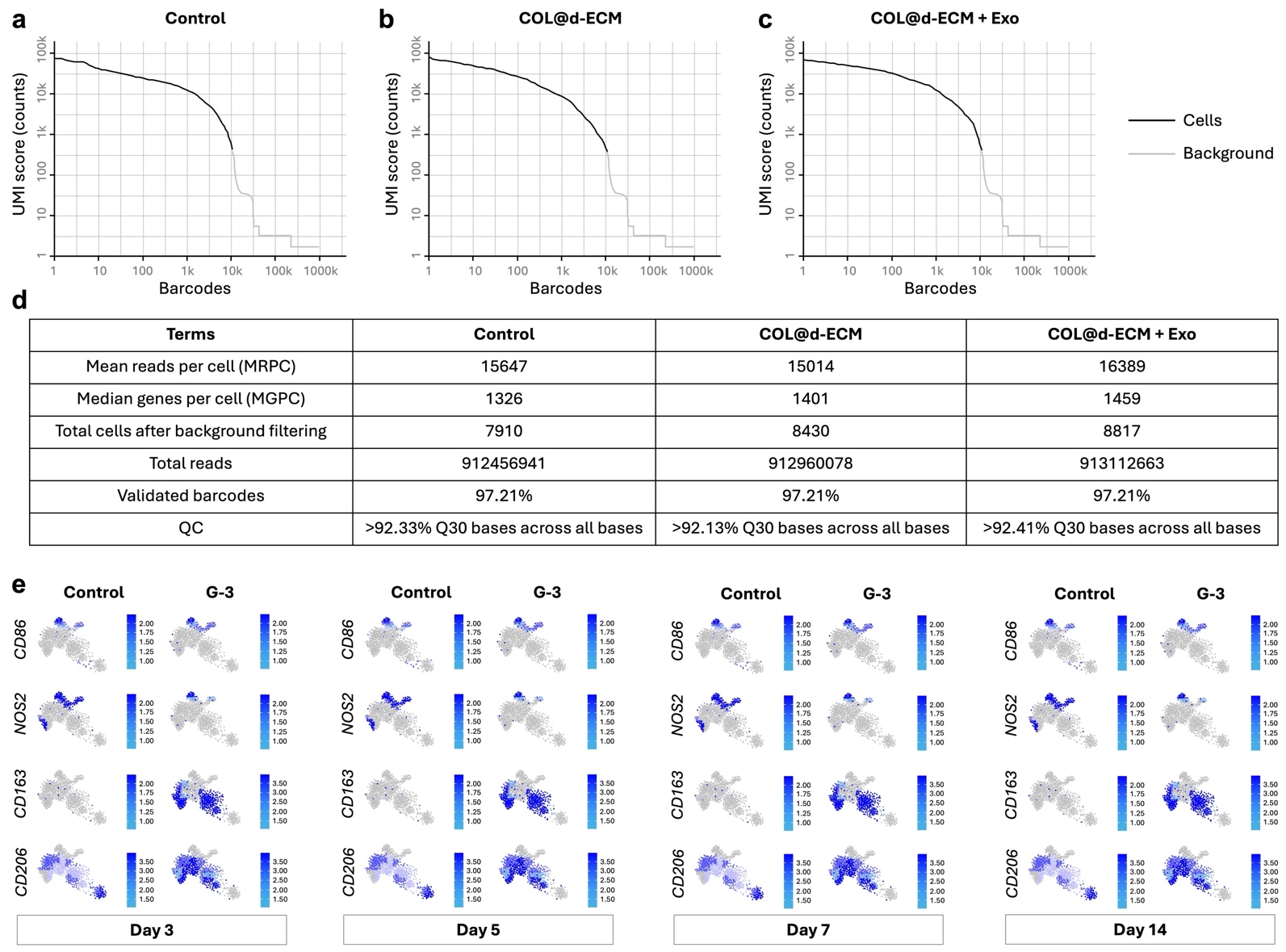


**Figure S15.** Quality control (QC) and analysis of scRNA-seq data. **(a-c)** Unique molecular identifiers (UMI) profiles of the various groups. **(d)** The summary of the QC in various groups, as predicted through CellRanger software (v3.1.0).

**Table S1.** A literature review on 3D bioprinting of functional skin grafts.

| **Biopolymer ink** | **Type of printing** | **Type of cells** | **References** |
| --- | --- | --- | --- |
| Fibrinogen | *In-situ* bioprinting | N/A | [[1](#_ENREF_1)] |
| 1. Dermal bioink – Collagen (3.5 mg/mL)  2. Epidermal bioink – Collagen (3.5 mg/mL) | DIW Bioprinting | Dermal fibroblast, Keratinocytes, Pericytes, and Endothelial cells | [[2](#_ENREF_2)] |
| 1. Dermal ink – (1% alginate + 5% gelatin)  2. Epidermal ink - (1% alginate + 5% gelatin) | DIW Bioprinting | Dermal fibroblast and Keratinocytes | [[3](#_ENREF_3)] |
| 1. Dermal ink – Fibrinogen (0.1 mg/mL) | DIW Bioprinting | Dermal fibroblast and Keratinocytes | [[4](#_ENREF_4)] |
| 10% bovine gelatin + 0.5% alginate | DIW Bioprinting | NIH 3T3 mouse fibroblast and human primary keratinocytes | [[5](#_ENREF_5)] |
| Tissue-specific collagen | DIW Bioprinting | Dermal fibroblasts, Melanocytes, and Keratinocytes | [[6](#_ENREF_6)] |
| Bioinks for full-thickness skin | DIW Bioprinting | Review | [[7](#_ENREF_7)] |
| Collagen | DIW Bioprinting | Dermal fibroblast | [[8](#_ENREF_8)] |
| Alginate + gelatin + diethylaminoethyl cellulose + fibrinogen | DIW Bioprinting | Dermal fibroblast and Keratinocytes | [[9](#_ENREF_9)] |
| 1. Dermal ink – 20% GelMA  2. Epidermal ink – 1.5% skin extracellular matrix (dECM) | DIW Bioprinting | Dermal fibroblast, Keratinocytes, and HUVECs | [[10](#_ENREF_10)] |
| Water soluble polyurethane | DIW Bioprinting | Dermal fibroblast and Keratinocytes | [[11](#_ENREF_11)] |
| 5% GelMA + 1.25% HAMA | DLP Bioprinting | Dermal fibroblast and HUVECs | [[12](#_ENREF_12)] |
| Polyethylene glycol diacrylate (HCC-PEG) | Multiphoton *in vivo* bioprinting | Direct skin printing  into the dermis region | [[13](#_ENREF_13)] |
| Alginate + gelatin + collagen | DIW bioprinting | Dermal fibroblasts | [[14](#_ENREF_14)] |
| Polycaprolactone + P407 | DIW bioprinting | Dermal fibroblasts | [[15](#_ENREF_15)] |
| No scaffold | Direct cell printing | Keratinocytes | [[16](#_ENREF_16)] |
| 5% GelMA + 8% collagen + Tyrosinase (0-800 U/mL) | DIW bioprinting | Dermal fibroblasts, Keratinocytes, and Melanocytes | [[17](#_ENREF_17)] |
| GelMA + phenylboric acid modified hyaluronic acid | 3D printing | L929 mouse fibroblasts | [[18](#_ENREF_18)] |
| **10% Chicken skin-derived d-ECM + 1% Rat tail Type-1 collagen**  **+ Exosomes** | **DIW bioprinting** | **hDFs (dermal layer),**  **hKCs (epidermal layer),**  **HUVECs (endothelial layer), biochemical and transcriptomic approach** | **This work** |

**Table S2.** List of antibodies and their dilutions used in this study.

| **Antibodies** | **Dilutions** | **Experiments** | **Company** |
| --- | --- | --- | --- |
| NOS2  (iNOS) | P (1:500)  S (1:500) | FACS (RAW 264.7) | Santa Cruz Biotechnology, USA |
| CD163 | P (1:500)  S (1:500) |  | Santa Cruz Biotechnology, USA |
| NOS2 | P (1:500)  S (1:250) | ICC (RAW 264.7) | Santa Cruz Biotechnology, USA |
| VEGFA | P (1:500)  S (1:250) |  | Santa Cruz Biotechnology, USA |
| CD163 | P (1:500)  S (1:250) |  | Santa Cruz Biotechnology, USA |
| CD9 | P (1:500)  S (1:500) | Western (exosomes) | Santa Cruz Biotechnology, USA |
| CD63 | P (1:500)  S (1:500) |  | Santa Cruz Biotechnology, USA |
| GAPDH | P (1:500)  S (1:500) |  | Santa Cruz Biotechnology, USA |
| CD31  (PECAM1) | P (1:500)  S (1:500) | IHC (3D bioprinted  scaffold) | Santa Cruz Biotechnology, USA |
| CD86 | P (1:1000)  S (1:500) | ICC (*in vivo* tissue  staining) | Santa Cruz Biotechnology, USA |
| NOS2 | P (1:1000)  S (1:500) |  | Santa Cruz Biotechnology, USA |
| CD163 | P (1:1000)  S (1:500) |  | Santa Cruz Biotechnology, USA |
| CD206 | P (1:1000)  S (1:500) |  | Santa Cruz Biotechnology, USA |

**Abbreviations:** *GAPDH*: Glyceraldehyde 3-phosphate dehydrogenase, *NOS2*: Nitric oxide synthase-1, *CD163*: Cysteine-rich family protein 163, *VEGFA*: Vascular endothelial growth factor A, *CD9*: Transmembrane 4 superfamily protein, *CD63*: Cell surface tetraspanin protein; *CD31*: Platelet endothelial cell adhesion molecule or PECAM1.

**Table S3.** Specified gene primers used for qRT-PCR analysis of mouse cells.

| **Genes** | **Froward primer (5′**$\to$**3′)** | **Reverse primer (3′**$\to$**5′)** |
| --- | --- | --- |
| *TNFα* | CCCTCACACTCAGATCATCTTCT | GCTACGACGTGGGCTACAG |
| *IL-1β* | TGCCACCTTTTGACAGTGATG | AAGGTCCACGGGAAAGACAC |
| *IL-4* | CCATATCCACGGATGCGACA | AAGCCCGAAAGAGTCTCTGC |
| *IL-10* | GCTCTTACTGACTGGCATGAG | CGCAGCTCTAGGAGCATGTG |
| *NOS2* | GCACATCAAAGCGGCCATAG | CGGCAAACATGACTTCAGGC |
| *VEGF* | GCAAGAGAAGACACGGTGGT | CAGGAGGTGGGGTAAGGAG |
| *Arg-1* | AACACGGCAGTGGCTTTAAC | GTCAGTCCCTGGCTTATGGTT |
| *TGF-β* | TGGAGCAACATGTGGAACTC | TGCCGTACAACTCCAGTGAC-3 |
| *STAT1* | CGCCAGAGAGAAATTCGTGT | TGAGATGTCCCGGATAGTGG |
| *STAT2* | CACTCCGCTTCCTCTATCC | CATCAATGGCAACTCCTG |
| *STAT3* | AGGAGTCTAACAACGGCAGCCT | GTGGTACACCTCAGTCTCGAAG |
| *STAT4* | TCAGTGAGAGCCATCTTGGAGG | TGTAGTCTCGCAGGATGTCAGC |
| *STAT6* | ACGACAACAGCCTCAGTGTGGA | CAGGACACCATCAAACCACTGC |
| *CD163* | GTGGTCAACTCCGCTTGGTA | CTTGGGGCACCATCTGTGAT |
| *IL-4R* | ACCAGATGGAACTGTGGGCTGA | AGCAGCCATTCGTCGGACACAT |
| *RTK* | GGACTGTGTCTCCTGCCAGAAT | GGCAGACATTCTGGATGGCACT |
| *GAPDH* | ACCACAGTCCATGCCATCA | TCCACCACCCTGTTGCTGT |

**Abbreviations:** *GAPDH*: Glyceraldehyde 3-phosphate dehydrogenase, *TNF-α*: Tumor necrosis factor alpha, *IL-1β*: Interleukin 1β, *IL-4*: Interleukin 4, *IL 10*: Interleukin 10, *NOS2*: Nitric oxide synthase-1, *VEGF*: Vascular endothelial growth factor, *Arg-1*: Arginase-1, *TGF-β*: Transforming growth factor-β, *STAT1-6*: Signal transducer and activator of transcription 1-6, *CD163*: Cysteine-rich family protein 163, *IL-4R*: Interleukin 4 receptor, *RTK*: Receptor tyrosine kinase.

**Table S4.** Specified gene primers used for qRT-PCR analysis of human cells.

| **Genes** | **Froward primer (5′**$\to$**3′)** | **Reverse primer (3′**$\to$**5′)** | **Cells** |
| --- | --- | --- | --- |
| *FN* | GCCATGACAATGGTGTGAAC | GCAAATGGCACCGAGATATT | hDFs |
| *COL1A* | CTGACCTTCCTGCGCCTGATGTCC | GTCTGGGGCACCAACGTCCAAGGG | hDFs |
| *KRT1* | AGAGTGGACCAACTGAAGAGT | ATTCTCTGCATTTGTCCGCTT | hKCs |
| *KRT5* | TGGTGCCGGTAGTGGATTTG | CAGGAGGGCAGACAGGAAAG | hKCs |
| *KRT14* | GATGATTGGCAGCGTGGAG | CAGAGGAGAACTGGGAGGAG | hKCs |
| *VEGF* | TATGCGGATCAAACCTCACCA | CACAGGGATTTTTCTTGTCTTGCT | hECs |
| *CD31* | GGCTCAGACATCCACATAACC | CTTACCAGGGCGTTCAGGGAC | hECs |
| *GAPDH* | ACCACAGTCCATGCCATCA | TCCACCACCCTGTTGCTGT | hDFs |

**Abbreviations:** *GAPDH*: Glyceraldehyde 3-phosphate dehydrogenase, *FN*: Fibronectin, *COL1A*: Collagen Type 1A, *KRT 1*: Keratin 1, *KRT 5*: Keratin 5, *KRT 14*: Keratin 14, *VEGF*: Vascular endothelial growth factor.

**Supplementary References:**

[1] Albanna M, Binder KW, Murphy SV, Kim J, Qasem SA, Zhao W, et al. In situ bioprinting of autologous skin cells accelerates wound healing of extensive excisional full-thickness wounds. *Scientific Reports*. 2019;9:1-15.

[2] Xie M, Kirkiles-Smith NC, Lee V, Hotta S, Dai G, Xu X, et al. 3D bioprinting of a vascularized and perfusable skin graft using human keratinocytes, fibroblasts, pericytes, and endothelial cells.

[3] Liu J, Zhou Z, Zhang M, Song F, Feng C, Liu H. Simple and robust 3D bioprinting of full-thickness human skin tissue. *Bioengineered*. 2022;13:10087-97.

[4] Cubo N, Garcia M, Del Canizo JF, Velasco D, Jorcano JL. 3D bioprinting of functional human skin: production and in vivo analysis. *Biofabrication*. 2016;9:015006.

[5] Pourchet LJ, Thepot A, Albouy M, Courtial EJ, Boher A, Blum LJ, et al. Human skin 3D bioprinting using scaffold‐free approach. *Advanced Healthcare Materials*. 2017;6:1601101.

[6] Ng WL, Qi JTZ, Yeong WY, Naing MW. Proof-of-concept: 3D bioprinting of pigmented human skin constructs. *Biofabrication*. 2018;10:025005.

[7] Daikuara LY, Chen X, Yue Z, Skropeta D, Wood FM, Fear MW, et al. 3D bioprinting constructs to facilitate skin regeneration. *Advanced Functional Materials*. 2022;32:2105080.

[8] Morley CD, Ellison S, Bhattacharjee T, O’Bryan CS, Zhang Y, Smith KF, et al. Quantitative characterization of 3D bioprinted structural elements under cell generated forces. *Nature Communications*. 2019;10:1-9.

[9] Ramakrishnan R, Kasoju N, Raju R, Geevarghese R, Gauthaman A, Bhatt A. Exploring the Potential of Alginate-Gelatin-Diethylaminoethyl Cellulose-Fibrinogen based Bioink for 3D Bioprinting of Skin Tissue Constructs. *Carbohydrate Polymer Technologies and Applications*. 2022:100184.

[10] Jin R, Cui Y, Chen H, Zhang Z, Weng T, Xia S, et al. Three-dimensional bioprinting of a full-thickness functional skin model using acellular dermal matrix and gelatin methacrylamide bioink. *Acta Biomaterialia*. 2021;131:248-61.

[11] Seol Y-J, Lee H, Copus JS, Kang H-W, Cho D-W, Atala A, et al. 3D bioprinted biomask for facial skin reconstruction. *Bioprinting*. 2018;10:e00028.

[12] Zhou F, Hong Y, Liang R, Zhang X, Liao Y, Jiang D, et al. Rapid printing of bio-inspired 3D tissue constructs for skin regeneration. *Biomaterials*. 2020;258:120287.

[13] Urciuolo A, Poli I, Brandolino L, Raffa P, Scattolini V, Laterza C, et al. Intravital three-dimensional bioprinting. *Nature Biomedical Engineering*. 2020;4:901-15.

[14] Niu C, Wang L, Ji D, Ren M, Ke D, Fu Q, et al. Fabrication of SA/Gel/C scaffold with 3D bioprinting to generate micro-nano porosity structure for skin wound healing: a detailed animal in vivo study. *Cell Regeneration*. 2022;11:1-12.

[15] Kang H-W, Lee SJ, Ko IK, Kengla C, Yoo JJ, Atala A. A 3D bioprinting system to produce human-scale tissue constructs with structural integrity. *Nature Biotechnology*. 2016;34:312-9.

[16] Madiedo-Podvrsan S, Belaïdi J-P, Desbouis S, Simonetti L, Ben-Khalifa Y, Collin-Djangone C, et al. Utilization of patterned bioprinting for heterogeneous and physiologically representative reconstructed epidermal skin models. *Scientific Reports*. 2021;11:1-12.

[17] Shi Y, Xing T, Zhang H, Yin R, Yang S, Wei J, et al. Tyrosinase-doped bioink for 3D bioprinting of living skin constructs. *Biomedical Materials*. 2018;13:035008.

[18] Feng Q, Li D, Li Q, Li H, Wang Z, Zhu S, et al. Assembling Microgels via Dynamic Cross-Linking Reaction Improves Printability, Microporosity, Tissue-Adhesion, and Self-Healing of Microgel Bioink for Extrusion Bioprinting. *ACS Applied Materials & Interfaces*. 2022;14:15653-66.
